# Genome-resolved metagenomics reveals microbiome diversity across 48 tick species

**DOI:** 10.1038/s41564-025-02119-z

**Published:** 2025-09-23

**Authors:** Li-Feng Du, Wenyu Shi, Xiao-Ming Cui, Huimin Fan, Jia-Fu Jiang, Cai Bian, Run-Ze Ye, Qian Wang, Ming-Zhu Zhang, Ting-Ting Yuan, Luo-Yuan Xia, Xiang-Dong Ruan, Qiao-Cheng Chang, Chun-Hong Du, Teng-Cheng Que, Xin Wang, Xiao-Hu Han, Tian-Ci Yang, Bao-Gui Jiang, Jian-Ying Chen, Xiao-Run Wang, Liang-Fei Tan, Yi-Wen Liu, Liang-Li Deng, Yan Liu, Yan Zhu, Yu-Sheng Pan, Ning Wang, Zhe-Tao Lin, Lian-Feng Li, Cheng Li, Shi-Jing Shen, Ya-Ting Liu, Di Tian, Xiao-Yu Han, Juan Wang, Yi-Fei Wang, Wan-Ying Gao, Yu-Yu Li, Tao Xiong, Tian-Hong Wang, Xiao-Yu Shi, Dai-Yun Zhu, Jin-Guo Zhu, Chong-Cai Wang, Wen-Qiang Shi, Lin Zhan, Zhi-Hong Liu, Dan Feng, Lin Zhao, Yi Sun, Tick Genome and Microbiome Consortium (TIGMIC), Li-Feng Du, Wenyu Shi, Xiao-Ming Cui, Huimin Fan, Jia-Fu Jiang, Run-Ze Ye, Qian Wang, Xiang-Dong Ruan, Qiao-Cheng Chang, Chun-Hong Du, Teng-Cheng Que, Xin Wang, Xiao-Hu Han, Tian-Ci Yang, Bao-Gui Jiang, Yan Liu, Jin-Guo Zhu, Chong-Cai Wang, Lin Zhan, Lin Zhao, Yi Sun, Jinfeng Wang, Na Jia, Fangqing Zhao, Wu-Chun Cao, Jinfeng Wang, Na Jia, Fangqing Zhao, Wu-Chun Cao

**Affiliations:** 1 State Key Laboratory of Pathogen and Biosecurity, Academy of Military Medical Sciences, Beijing, People’s Republic of China; 2 Institute of EcoHealth, School of Public Health, Shandong University, Jinan, People’s Republic of China; 3 Ministry of Education Key Laboratory of Contemporary Anthropology, School of Life Sciences, Fudan University, Shanghai, People’s Republic of China; 4 State Key Laboratory of Animal Biotech Breeding, College of Biological Sciences, China Agricultural University, Beijing, People’s Republic of China; 5 Research Unit of Discovery and Tracing of Natural Focus Diseases, Chinese Academy of Medical Sciences, Beijing, People’s Republic of China; 6 College of Food Science and Nutritional Engineering, China Agricultural University, Beijing, People’s Republic of China; 7 Mudanjiang Forestry Central Hospital, Mudanjiang, People’s Republic of China; 8 Department of Emergency Medicine, Qilu Hospital of Shandong University, Jinan, People’s Republic of China; 9 Shandong Provincial Hospital Affiliated to Shandong First Medical University, Jinan, People’s Republic of China; 10 School of Medicine, Nankai University, Tianjin, People’s Republic of China; 11 Academy of Forest Inventory and Planning, State Forestry and Grassland Administration, Beijing, People’s Republic of China; 12 School of Public Health, Shantou University, Shantou, People’s Republic of China; 13 Yunnan Institute for Endemic Diseases Control and Prevention, Dali, People’s Republic of China; 14 Faculty of Data Science, City University of Macau, Macau, People’s Republic of China; 15 Qingjiangpu District Center for Disease Control and Prevention, Huai’an, People’s Republic of China; 16 Shenyang Agriculture University, Shenyang, People’s Republic of China; 17 State Key Lab of Mosquito-borne Diseases, Hangzhou International Tourism Healthcare Center, Hangzhou Customs of China, Hangzhou, People’s Republic of China; 18 Lhasa Animal Epidemic Disease Prevention Control Center, Lhasa, People’s Republic of China; 19 Qinghai Animal Center for Disease Prevention and Control, Xining, People’s Republic of China; 20 Hubei Center for Disease Control and Prevention, Wuhan, People’s Republic of China; 21 Wuwei Center for Disease Prevention and Control, Wuwei, People’s Republic of China; 22 Chengdu Center for Disease Prevention and Control, Chengdu, People’s Republic of China; 23 Department of Microbiology, School of Basic Medica, Anhui Medical University, Hefei, People’s Republic of China; 24 Qinghai International Travel Health Care Center, Xining, People’s Republic of China; 25 Ningxia Medical University, Yinchuan, People’s Republic of China; 26 Shaanxi Provincial Center for Disease Control and Prevention, Xi’an, People’s Republic of China; 27 ManZhouLi Customs District, Manzhouli, People’s Republic of China; 28 Hainan International Travel Healthcare Center, Haikou, People’s Republic of China; 29 Central Laboratory, Guizhou Provincial People’s Hospital, Guiyang, People’s Republic of China; 30 Hospital Management Institute, Department of Innovative Medical Research, Chinese PLA General Hospital, Beijing, People’s Republic of China; 31 Institute of Zoology, Chinese Academy of Sciences, Beijing, People’s Republic of China; 32 University of Chinese Academy of Sciences, Beijing, People’s Republic of China; 33 Key Laboratory of Systems Biology, Hangzhou Institute for Advanced Study, University of the Chinese Academy of Sciences, Chinese Academy of Sciences, Hangzhou, People’s Republic of China

**Keywords:** Pathogens, Metagenomics, Bacterial genetics

## Abstract

Ticks are arthropod vectors capable of transmitting a wide spectrum of pathogens affecting humans and animals. However, we have relatively limited information of their genomic characteristics and the diversity of associated microbiomes. Here we used long- and short-read sequencing on 1,479 samples from 48 tick species across eight genera from China to determine their genome and associated pathogens and microbiome. Through de novo assembly, we reconstructed 7,783 bacterial genomes representing 1,373 bacterial species, of which, 712 genomes represented 32 potentially pathogenic species. Computational analysis found nutritional endosymbionts to be prevalent and highly specific to tick genera. The microbiome genome-wide association study revealed host genetic variants linked to pathogen diversity, abundance and key biological pathways essential to tick biology, including blood-feeding and pathogen invasion. These findings provide a resource for studying the host–microbe interactions within ticks, paving the way for strategies to control tick populations and tick-borne diseases.

## Abstract (web summary)

Sequencing the genome and microbiome of about 1,500 tick samples from regions across China revealed host–microbe associations in ticks that could have implications for controlling ticks and tick-borne diseases.

## Main

Ticks are obligate blood-feeding ectoparasites that transmit pathogens to humans. As vectors, ticks are capable of spreading a diverse array of bacterial, viral and parasitic pathogens to both humans and animals^1,2^. The geographical expansion and global spread of certain tick populations^3–7^, along with the increasing prevalence of endemic and emerging tick-borne diseases^8–11^, have raised substantial concerns for public and animal health worldwide.

Ticks harbour complex microbial communities due to their widespread distribution, unique feeding behaviour, diverse range of animal hosts and remarkable adaptability to various environments^12,13^. Microbiome studies on certain tick species have revealed substantial bacterial diversity^14–22^, providing valuable taxonomic insights into specific tick species. However, the microbiomes of most tick species remain largely unexplored. A systematic investigation of large-scale samples covering various tick genera and species across different eco-geographies, feeding statuses and other ecological traits is urgently needed. Our previous research highlighted the multifactorial influences of ecogeographical factors and tick genetic variations on the distribution of tick-borne bacterial pathogens^23^. However, to fully elucidate the complex tripartite interactions among ticks, pathogens and microbiota, comprehensive studies encompassing a broad range of tick species and environmental niches are essential.

In this Article, we used both short-read (Illumina) and long-read (nanopore) sequencing technologies to conduct a large-scale hologenomic analysis of 1,479 samples (1,460 processed via Illumina sequencing and 19 via nanopore sequencing) from 48 tick species across China. Our objectives were to explore the diversity of tick-associated bacteria and the factors influencing their composition, identify potential tick-borne pathogens, investigate the role of bacterial endosymbionts and their relationships with ticks and pathogens, and elucidate the link between tick genetic variability and pathogen carriage. By leveraging this extensive dataset, we provide insights into the tripartite interactions among ticks, tick-borne pathogens and microbiota, aiming to advance strategies for the control of ticks and tick-borne infections in both humans and animals.

## Results

### Sequencing of 1,479 samples covering representative tick species

**Fig. 1:**
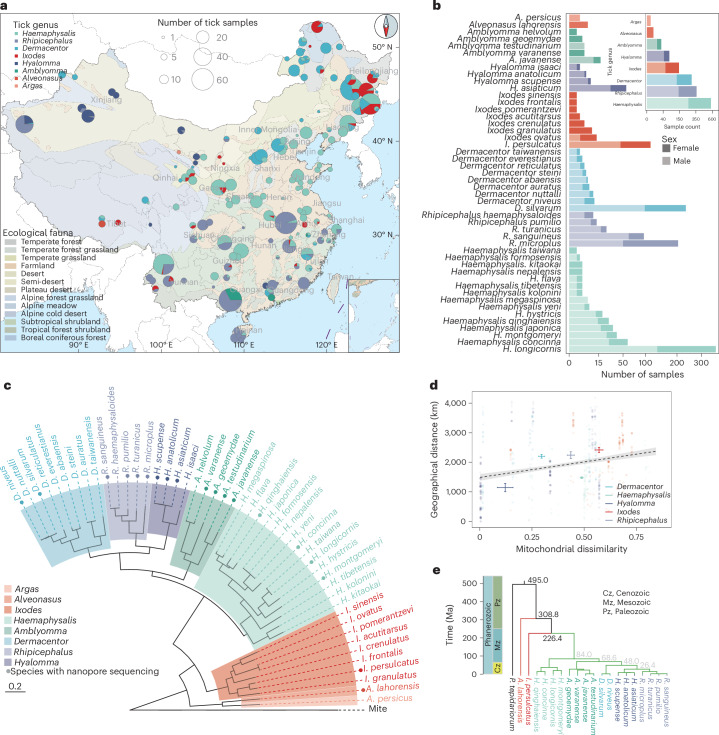
Sample distribution and evolutionary relationship of ticks. **a**, Map of collection of samples. The size of the circle represents the number of tick samples collected in the area, and the colours represent different genera of ticks. Different backgrounds on the map represent different ecological faunas. **b**, Number of samples of different tick species and genus. Dark colours indicate female ticks, while light colours represent male ticks. The principal representative species for each tick genus are as follows: *H. longicornis* for *Haemaphysalis*, *D. silvarum* for *Dermacentor*, *R. microplus* for *Rhipicephalus*, *I. persulcatus* for *Ixodes*, *H. asiaticum* for *Hyalomma* and *A. javanense* for *Amblyomma*. **c**, Phylogenetic tree of tick species based on the mitochondrial genome with mite as outgroup. Different background colours represent different tick genera, while dots indicate tick species that have undergone nanopore sequencing. **d**, Correlation between mitochondrial dissimilarity and geographical distance (km) within each genus. The vertical axis represents the geographical distance between samples, while the horizontal axis represents the mitochondrial dissimilarity among tick species represented by the samples. The crosses represent the 99% confidence intervals for mitochondrial dissimilarity (*x* axis) and geographical distance (*y* axis), centred on the mean values calculated within each genus. The colours of different points represent different genera of ticks. The dashed line represents the Pearson’s correlation between them (*r* = 0.24, *P* = 3.06 × 10^−15^, two-sided, *n* = 1,020), with the shaded error band indicating the 95% confidence interval around the estimated mean trend based on all individual samples. **e**, Phylogenetic tree of 19 tick genomes obtained in this study with *P. tepidariorum* as outgroup based on 350 single copy orthologues. The estimated divergence time between clades is labelled on the branch nodes. Geological eras are marked on the right side of the timeline. Ma, million years ago.

**Extended Data Fig. 1:**
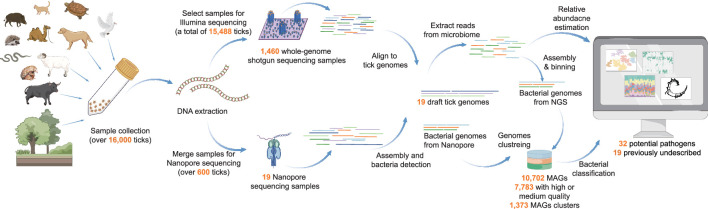
Flowchart of study design. Figure created with Figdraw.com.

To comprehensively characterize tick genomes and their associated microbiomes, we collected over 16,000 individual adult ticks representing 48 tick species across 8 genera from all 31 provinces, municipalities and autonomous regions of mainland China, encompassing diverse ecological faunas (Fig. 1a). Individual ticks were pooled into 1,460 samples based on species, sex, collection site and blood-feeding status, with each pooled sample undergoing whole-genome shotgun sequencing (Extended Data Fig. 1 and Supplementary Tables 1–4). In each genus of ticks, there is a primary representative species (Fig. 1a,b).

To explore the potential relationship between host genetic differentiation and adaptation to ecological environments in connection with the tick microbiome, we first de novo assembled the mitochondrial genomes of all 48 tick species from the whole-genome sequencing data. Mitochondria-based phylogeny corroborated the morphological classification of each tick species (Fig. 1c and Supplementary Note 1), reinforcing the accuracy of host background information in this study. To further explore the connection between geographic distribution and genetic evolution, we analysed genetic distances among the 1,460 samples relative to their geographical separations. Pearson’s correlation revealed a positive relationship between geographical distance and genetic divergence, with greater genetic divergence observed over larger distances (*r* = 0.24, *P* = 3.06 × 10^−15^; Fig. 1d), suggesting genetic evolutionary variations in ticks may play a crucial role in their adaptation to diverse ecological environments^24^.

As nucleic acids were extracted from whole ticks, it is essential to eliminate host-derived sequence interference in the metagenomic data. Currently, among the tick species distributed in China, only six have published genome assemblies, which were shown in our previous study^23^. To compensate for the lack of genomic information, we pooled samples of each tick species collected from various sites for nanopore sequencing (Extended Data Fig. 1). Through de novo assembly, we successfully generated draft genomes for 19 tick species^25^ (Supplementary Table 5), with 13 of them being newly sequenced (Fig. 1e). The 19 tick genomes provided useful levels of genome completeness (81 ± 11%) but low contiguity (N50, the length of the shortest contig for which longer and equal length contigs cover 50% of the assembly, = 70 ± 61 kilobases (Kb)), possibly due to low coverage sequencing and high within-species variability (Supplementary Note 2). Leveraging these draft genomes, we effectively filtered out tick sequences, retaining a median of 15.6% of Illumina reads per sample for microbiome analysis. This approach also facilitated the identification of 33 circular bacterial genomes from the nanopore sequencing data, establishing a critical foundation for subsequent microbiome investigations.

### Discovery of 7,783 bacterial genomes

By integrating short-read and long-read sequencing data, we used a de novo metagenome assembly pipeline^26,27^, and a total of 10,702 metagenome-assembled genomes (MAGs) were obtained. Subsequently, after a quality control step, we focused on 7,783 MAGs with medium quality or better, meeting criteria of at least 50% completeness and less than 10% contamination as Genomic Standards Consortium recommended^28^. The mean and median sequencing coverage depth per assembly were 42.3× and 28.0×. Based on the average nucleotide identity (ANI) calculation between these MAGs, 1,373 clusters were generated (Supplementary Table 6).

**Fig. 2:**
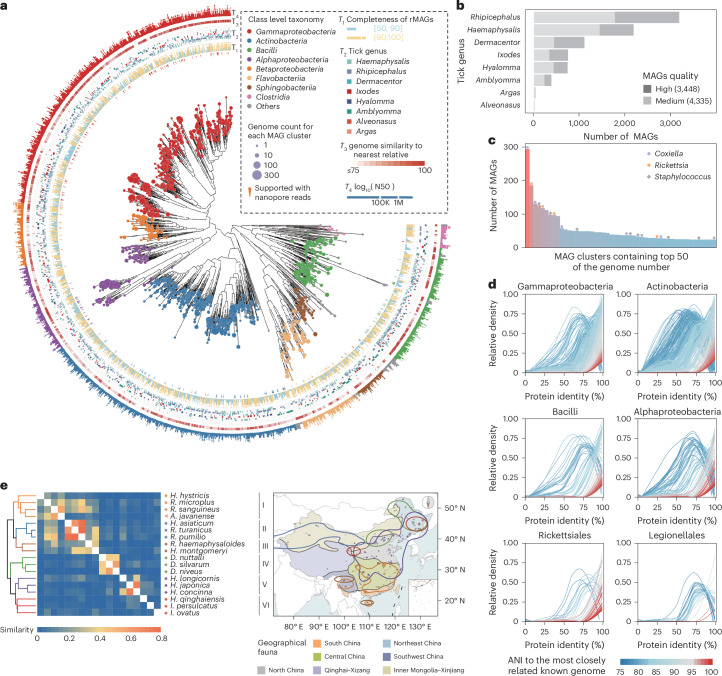
Features of MAGs and their relationship with ticks. **a**, Maximum-likelihood phylogenetic tree of the 1,373 rMAGs assembled in this study. The size of the leaf node represents the genome counts for each MAG cluster, and the colour of the leaf node represents the taxonomy of rMAGs at the class level. The triangular markers indicate that the rMAGs are supported by nanopore sequencing data. From the inner rings to the outer rings, four types of information: completeness (*T*_1_), genus of tick samples (*T*_2_), ANI with most similar genome in public (*T*_3_) and N50 (*T*_4_) are labelled. 100K, 100 thousand; 1M, 1 million. **b**, The counts of bacterial genomes assembled from each tick genus. High-quality (completeness ≥ 95% and contamination < 5%) genomes are indicated in dark grey, while medium-quality (completeness ≥ 50% and contamination < 10%) genomes are shown in light grey. **c**, The count of genomes in MAG clusters containing top 50 counts. Each column represents a MAG cluster. Top three frequent genera *Coxiella*, *Rickettsia* and *Staphylococcus* were marked with dots of different shapes. **d**, The distribution of protein similarity between proteins identified in rMAGs and the most similar proteins in the NCBI non-redundant (NR) protein sequence database is displayed. The rMAGs annotated as Gammaproteobacteria, Actinobacteria, Bacilli, Alphaproteobacteria, Rickettsiales and Legionellales are showcased. **e**, Overall microbial carriage similarity among ticks with sample counts over 10. In the similarity heat map, orange indicates high similarity in microbiome carriage, while blue indicates low similarity.

We selected the highest quality representative MAGs (rMAGs) from each cluster to construct a maximum likelihood phylogenetic tree. The 1,373 rMAGs were predominantly classified into eight bacterial classes (Fig. 2a). Among the tick genera analysed, we obtained 2,952 bacterial genomes from 351 *Rhipicephalus* samples and 2,025 bacterial genomes from *Haemaphysalis*. The genus *Argas*, sampled from only two samples, yielded 32 bacterial genomes (Fig. 2b).

It is worth noting that among the top 50 clusters, genomes of *Coxiella* and *Coxiella*-like bacteria were the most predominant, followed by *Rickettsia*, both of which are well recognized as tick-associated bacteria (Fig. 2c). Of the 1,373 rMAGs, approximately two-thirds were tentatively classified as undefined species, as determined by ANI values below 95% (ref. ^29^). Protein sequences from rMAGs with ANI above 95% showed nearly 100% similarity to known bacteria in the NCBI non-redundant (NR) protein sequence database^30^, whereas those from the remaining 908 rMAGs were highly distinct from any known bacterial species (Fig. 2d). These findings highlight the vastly unexplored diversity of the tick-associated microbiota, emphasizing the potential for previously undescribed bacterial species yet to be characterized.

Subsequently, we identified the host- and region-specific characteristics of the tick microbiome by analysing variations in microbial communities across different tick genera, species and geographical regions. By comparing the microbial carriage similarity among 18 tick species with sample sizes exceeding 10, we identified six distinct clusters based on microbial composition patterns^31^. Clusters I to III appear to be predominantly influenced by geographic distribution, whereas the composition of clusters IV and V may be largely explained by their shared taxonomic lineage (Fig. 2e and Supplementary Note 3). These findings underscore both geographic and taxonomic influences in shaping tick-associated microbiomes, providing key insights into the intricate relationships between ticks, their microbial communities and environmental factors.

### Characterization of tick microbiome according to ecotypes

**Fig. 3:**
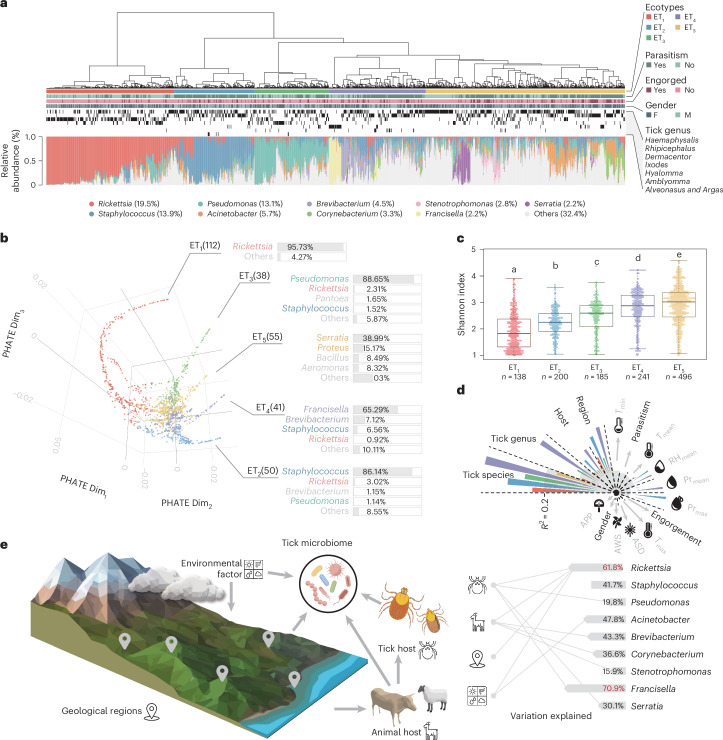
Ecotypes of tick microbiomes and their characteristics. **a**, Hierarchical clustering of tick microbiome samples and the corresponding ecotype assignment, tick host information and taxonomic profiles. The sample information from top to bottom includes ecotype, parasitized statuses, engorgement, gender and tick genus. The top nine genera of highest abundance are displayed. F, female; M, male. **b**, Potential of Heat-Diffusion for Affinity-Based Transition Embedding (PHATE) dimension reduction results for tick microbiome samples. Dim_*n*_, dimension. The number following each ecotype designated as ET_*n*_ indicates the count of representative samples within each ecotype. The average relative abundance of microbial genera is displayed for representative samples. **c**, Alpha diversity of samples corresponding to the five ecotypes. In each box plot, the centre line indicates the median, the edges of the box represent the first and third quartiles, and the whiskers extend to span a 1.5 interquartile range from the edges. Different letters indicate statistically significant differences at *P* < 0.05 by analysis of variance. **d**, Correlations between different ecotypes and overall sample characteristics (including tick, animal hosts, geographic locations and environmental factors) were determined using permutation tests. Five colours represent five different ecotypes, while grey represents the overall sample. The abbreviations are as follows: *T*_min_, mean minimum temperature; *T*_mean_, mean temperature; RH_mean_, mean relative humidity; Pr_mean_, mean daily precipitation; Pr_max_, maximum precipitation; *T*_max_, mean maximum temperature; ASD, monthly average sunshine duration; AWS, average wind speed per 2 min; AAP, average atmospheric pressure. The grey text represents environmental factors, while the black text indicates characteristics of ticks. **e**, The model of environmental influences on tick microbiome and significant factors affecting high-abundance genera. Link diagram on the right shows the factors influencing the specific microbial taxa using the random forest model. Panel **e** created with Figdraw.com.

**Extended Data Fig. 2:**
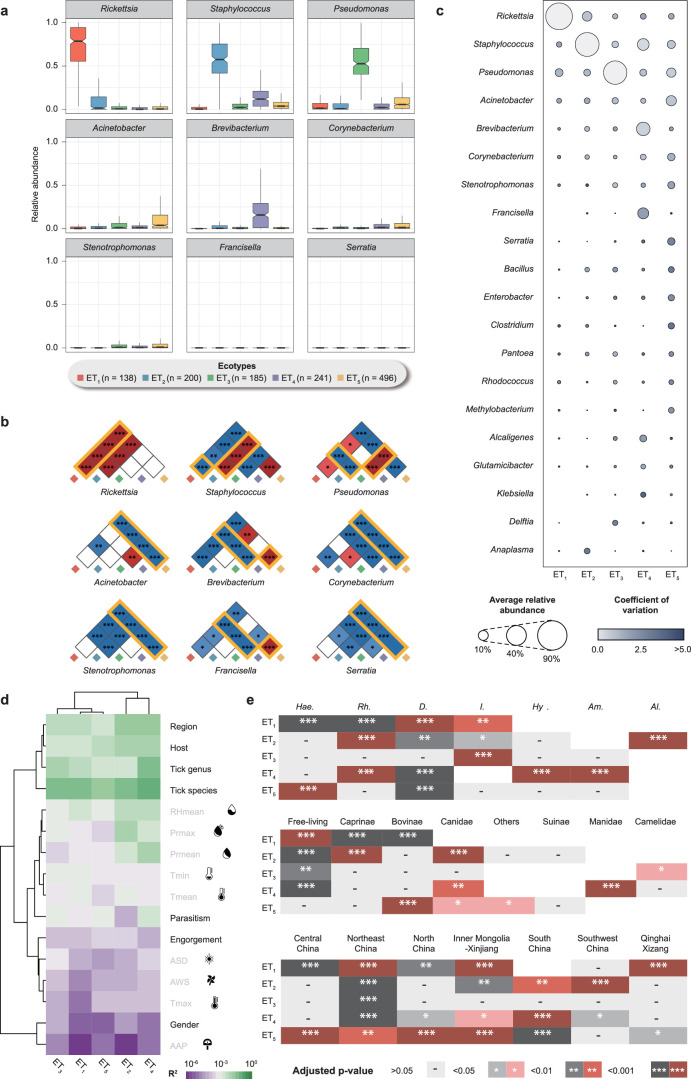
Differences in bacteria among ecotypes and influencing factors. (**a**) Differences in abundance of the nine high-abundance genera across different ecotypes. Box plots show the median (center line), the 25th and 75th percentiles (box limits), and whiskers extending to values within 1.5× interquartile range. (**b**) Statistical significance levels of the nine high-abundance genera across different ecotypes. Comparisons are made between the former to the latter ecotype. Red squares indicate the former is significantly higher than the latter and the blue indicate the former is significantly lower than the latter (two-sided t-test, *: P-value < 0.05, **: P-value < 0.01, ***: P-value < 0.001, P-values were adjusted by Benjamini-Hochberg method). The yellow boxes represent the enrichment of the nine high-abundance genera across different ecotypes. (**c**) Relative abundance (size) and coefficient of variation (color) of major bacterial species across different ecotypes. (**d**) Hierarchical clustering of influencing factors across different ecotypes. (**e**) Associations among ecotypes and various factors across categories. Red and black indicate enrichment and depletion, respectively (Chi-square test, -: P-value > 0.05, *: P-value < 0.05, **: P-value < 0.01, ***: P-value < 0.001).

To delve deeper into the factors shaping the tick microbiome, we performed taxonomic profiling to examine the relationships between microbial composition and ecogeographical factors, animal hosts and biological traits of ticks. We introduced the synonymous term ‘ecotype’ (ET) to categorize tick microbiomes based on their microbial abundance profiles and investigate the characteristics and relationships with these classifications. Five distinct tick microbiome ecotypes were identified (Fig. 3a), each dominated by specific bacterial taxa (Fig. 3b, Extended Data Fig. 2a and Supplementary Note 4). Significant variations in the proportions of dominant bacteria were observed across ecotypes (Extended Data Fig. 2b), with median proportions exceeding 50% in ET_1_, ET_2_ and ET_3_ (Extended Data Fig. 2c). Alpha diversity differed markedly among ecotypes, showing a progressive increase from ET_1_ to ET_5_ (Fig. 3c).

To examine the influence of ticks, their hosts, habitats and environmental conditions on tick-associated microbial communities, we used permutational multivariate analysis of variance (PERMANOVA) based on Bray–Curtis distances between samples^32^. It is worth noting that environmental factors related to humidity and temperature exerted significant influences on the microbiome. Across different ecotypes, ET_2_ and ET_4_ showed similar patterns of environmental influence, particularly with respect to tick species, host, region, mean relative humidity, mean daily precipitation and maximum precipitation (Fig. 3d and Extended Data Fig. 2d), indicating shared environmental drivers or interactions shaping microbial community dynamics.

To further explore how tick species, hosts and geographical locations correlate with the ecotypes of tick microbial communities, we used chi-square tests to assess the associations between ecotypes and factors. ET_1_ showed a notable positive correlation with specific tick species, their free-living status and their prevalence in Northeast China and Inner Mongolia–Xinjiang region (Extended Data Fig. 2e). As shown in Fig. 3a, ET_1_ was characterized by a dominance of *Rickettsia*, which are vertically transmitted, thereby reducing the reliance on bacterial acquisition from animal hosts. This underscores the importance of free-living status as a key determinant for ET_1_.

**Extended Data Fig. 3:**
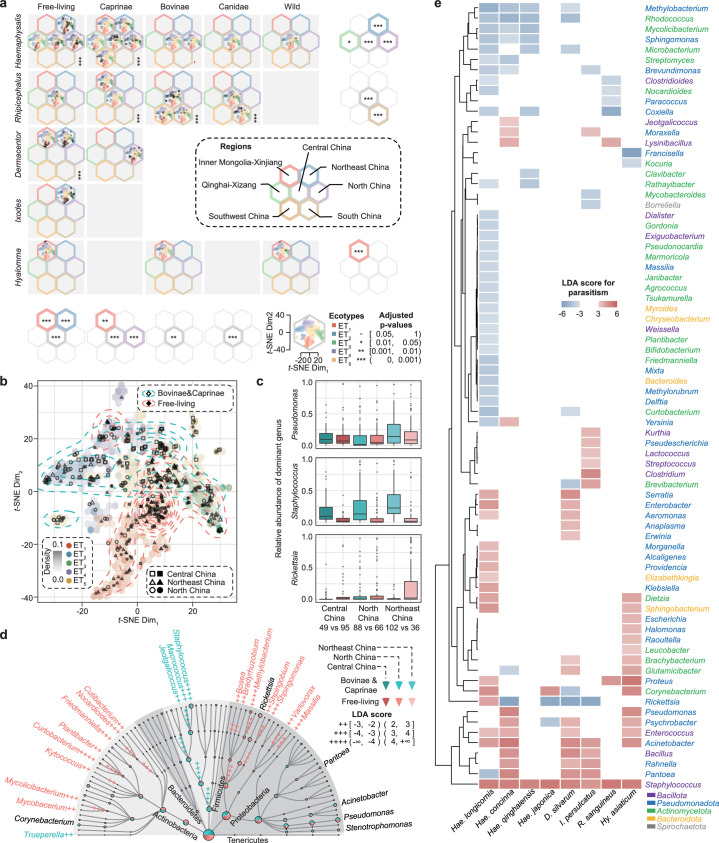
Relationships between ecotypes and geography, tick species, and animal hosts. . (**a**) The t-SNE dimensionality reduction distribution of tick microbiome data under three factors. Unsupervised clustering and dimensionality reduction of the microbiome taxonomic profile using t-distributed stochastic neighbor embedding (t-SNE) in the bottom right corner. Each hex cell represents a specific region of China, each row represents a specific tick host, while each column corresponds to a specific animal host, points inside each hex cell indicate tick samples, and the five background colors denote five ecotypes. A series of PERMANOVA were conducted. When comparing by row, the tick genus is fixed, and the P-value in the hex cell at the end of each row represents a fixed tick genus and geographic location of tick samples. When comparing by column, the animal host is fixed, and the P-value in the hex cell at the end of each column indicates a fixed animal host and geographic area of tick samples, comparing differences between various tick genus. (**b**) Detailed dimensionality reduction plot of the microbiomes using t-SNE method in comparison between free-living ticks and those parasitizing on cattle or sheep. The color of the hexagons indicates different ecotypes, while the shade of the color represents sample density. Different shapes of points indicate different geographic regions, with solid and hollow points representing free-living and cattle/sheep-parasitic samples, respectively. (**c**) Relative abundance of the dominant bacterial genus in free-living and cattle/sheep-parasitic ticks across different regions. Red indicates free-living ticks, while green represents samples parasitizing cattle and sheep. Box plots show the median (center line), the 25th and 75th percentiles (box limits), and whiskers extending to values within 1.5× interquartile range. Outliers beyond this range are shown as individual points. (**d**) Differences in microbiome at different bacterial taxonomy levels between free-living ticks and those parasitizing cattle and sheep across various geographic regions. The size of the circle indicates the relative abundance. A linear discriminant effect size analysis (LEfSe) was used at different taxonomical levels to find the genera representing parasitism difference. (**e**) Differences in bacterial communities between parasitic and free-living ticks across various tick species. Blue denotes bacteria enriched in free-living ticks, while red indicates bacteria enriched in parasitic ticks. The intensity of the color represents the Linear Discriminant Analysis (LDA) score.

In addition, to analyse the interplay between tick species, geographical regions and hosts on microbial communities, we systematically controlled for two of these factors and evaluated the contribution of the remaining factor to microbiome variation (Extended Data Fig. 3a). The results indicated that when ticks fed on cows and were parasitized, geographical regions did not substantially influence microbial communities. However, all other comparisons revealed substantial differences, highlighting substantial variability in tick microbiomes under the combined influence of these factors. Using the most abundant genus, *Haemaphysalis*, as a case study, we investigated the impacts of regional and host factors on microbiome composition. Host-attached samples showed a higher probability of their microbial communities being classified as ET_2_ or ET_4_, whereas free-living samples harboured greater abundances of Actinobacteria, α-proteobacteria and β-proteobacteria. Ticks parasitizing breeds of cattle or sheep were predominantly associated with bacteria from the Staphylococcaceae family (Extended Data Fig. 3c,d). Significant differences in microbial composition were observed between parasitized and free-living ticks across various tick species (Extended Data Fig. 2e). Random forest analysis further reinforced that tick species and animal hosts exerted the strongest influence on microbiome composition (Fig. 3e). Among them, *Rickettsia* showed a dynamic response to multiple environmental and biological factors, while both *Rickettsia* and *Francisella* exceed 60%, indicating that their distribution patterns were strongly influenced by environmental variables (Supplementary Table 7).

### Taxonomic and functional diversity of tick-associated bacteria

**Extended Data Fig. 4:**
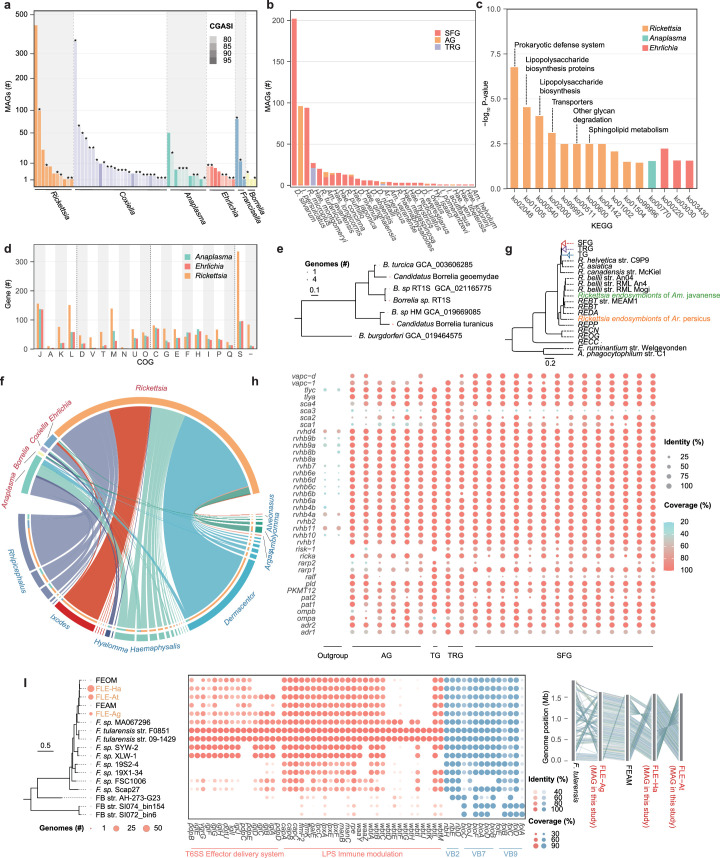
Genomic characteristics of tick-associated bacteria. . (**a**) Number of MAGs in this study. Different colors represent different genera. Each bar represents a species, with an asterisk (*) indicating genomes not present in public databases. (**b**) Number of rickettsial genomes, categorized by *Rickettsia* groups. (**c**) Pathways enriched in genus-specific genes within *Anaplasma*, *Ehrlichia*, and *Rickettsia*, and their significance levels according to Kyoto Encyclopedia of Genes and Genomes (KEGG). (**d**) Clusters of Orthologous Groups (COG) annotations of genus-specific genes in *Anaplasma*, *Ehrlichia*, and *Rickettsia*, with different colors representing different genera. J: Translation, ribosomal structure and biogenesis; A: RNA processing and modification; K: Transcription; L: Replication, recombination and repair; B: Chromatin structure and dynamics; D: Cell cycle control, cell division, chromosome partitioning; Y: Nuclear structure; V: Defense mechanisms; T: Signal transduction mechanisms; M: Cell wall/membrane/envelope biogenesis; N: Cell motility; Z: Cytoskeleton; W: Extracellular structures; U: Intracellular trafficking, secretion, and vesicular transport; O: Posttranslational modification, protein turnover, chaperones; C: Energy production and conversion; G: Carbohydrate transport and metabolism; E: Amino acid transport and metabolism; F: Nucleotide transport and metabolism; H: Coenzyme transport and metabolism; I: Lipid transport and metabolism; P: Inorganic ion transport and metabolism; Q: Secondary metabolites biosynthesis, transport and catabolism; R: General function prediction only; S: Function unknown. (**e**) Phylogenetic tree of *Borrelia* genomes. Reference genomes are represented by gray dots. (**f**) Distribution of pathogens across different tick species in this study. The thickness of the chord represents the number of positive samples. Different colors represent different genera of ticks and the tick species are arranged from left to right as follows: *R. microplus*, *R. turanicus*, *R. pumilio*, *R. haemaphysaloides*, *R. sanguineus*, *I. persulcatus*, *I. pomerantzevi*, *I. ovatus*, *I. granulatus*, *I. acutitarsus*, *Hy. asiaticum*, *Hy. isaaci*, *Hae. montgomeryi*, *Hae. longicornis*, *Hae. concinna*, *Hae. japonica*, *Hae. qinghaiensis*, *Hae. hystricis*, *Hae. megaspinosa*, *Hae. tibetensis*, *Hae. yeni*, *Hae. kolonini*, *Hae. flava*, *D. silvarum*, *D. niveus*, *D. abaensis*, *D. nuttalli*, *D. reticulatus*, *D. everestianus*, *Ar. persicus*, *Am. javanense*, *Am. varanense*, *Am. geoemydae*, *Am. helvolum* and *Al. lahorensis*. (**g**) Phylogenetic tree of *Rickettsia* primarily annotated as endosymbionts. Species names are abbreviated as follows: *Rickettsia* endosymbiont of *Bemisia tabaci*: REBT; *Rickettsia* endosymbiont of *Diachasma alloeum*: REDA; *Rickettsia* endosymbiont of *Pyrocoelia pectoralis*: REPP; *Rickettsia* endosymbiont of *Culicoides newsteadi*: RECN; *Rickettsia* endosymbiont of *Oedothorax gibbosus*: REOG; *Rickettsia* endosymbiont of *Cardiosporidium cionae*: RECC. (**h**) Pathogenic features of *Rickettsia* genomes. The size of the dots indicates coverage, while the shade of the color represents similarity. (**I**) Evolutionary relationships of *Francisella* MAGs assembled in this study, showcasing the identity (color) and coverage (size) of virulence and nutritional genes, along with genomic collinearity. The calculation method for coverage is the length of the consistent region divided by the full length of the reference gene. The species names are abbreviated as follows: Francisellaceae bacterium (FB), *Francisella*-like endosymbiont of *Amblyomma geoemydae* (FLE-Ag), *Francisella* endosymbiont of *Amblyomma maculatum* (FEAM), *Francisella*-like endosymbiont of *Amblyomma testudinarium* (FLE-At), *Francisella* endosymbiont of *Ornithodoros moubata* (FEOM), and *Francisella*-like endosymbiont of *Hyalomma anatolicum* (FLE-Ha).

The tick microbiome is highly complex, consisting primarily of environmental bacteria and tick-associated bacteria^13^. The latter typically include species from the genera *Rickettsia*, *Coxiella*, *Anaplasma*, *Ehrlichia*, *Francisella* and *Borrelia*. To explore this complexity, we extracted genomes annotated to these six genera from 1,373 rMAGs and identified 11 *Rickettsia* species encompassing 582 MAGs, 26 *Coxiella* species with 624 MAGs, 11 *Anaplasma* species with 95 MAGs, 8 *Ehrlichia* species with 30 MAGs, 3 *Francisella* species with 89 MAGs and 3 *Borrelia* species with 5 MAGs. By comparing these genomes to those of known bacteria, we identified six *Rickettsia* species with 111 MAGs, seven *Anaplasma* species with 38 MAGs, six *Ehrlichia* species with 20 MAGs and two *Borrelia* species with 4 MAGs, all of which had not been previously characterized (Extended Data Fig. 4a). While 24 out of 26 *Coxiella* species and all *Francisella* species were initially classified as undefined bacteria, based on the current understanding of tick-borne microbes^33,34^, we have reclassified them as *Coxiella*-like endosymbionts (*Coxiella*-LE) and *Francisella*-like endosymbionts (*Francisella*-LE).

**Fig. 4:**
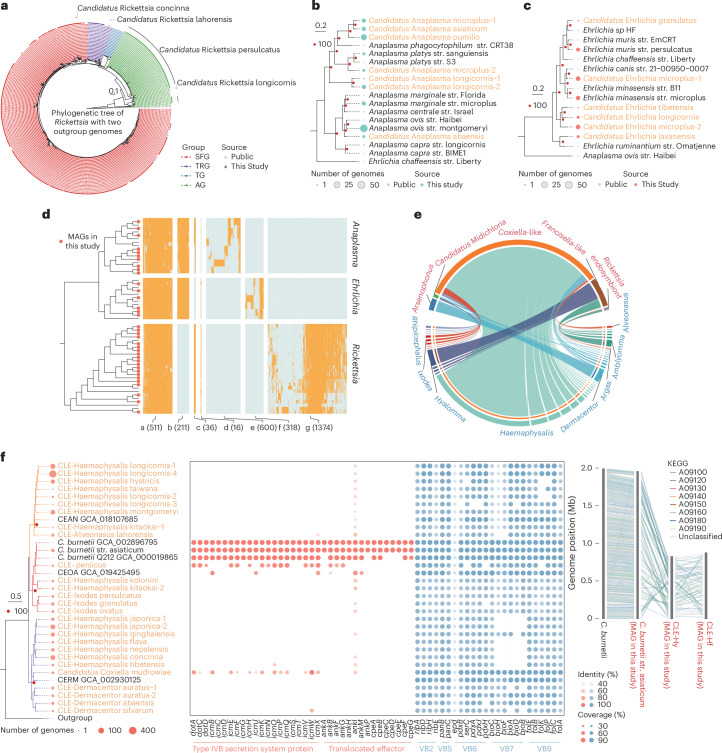
Diversity of tick-associated bacteria. **a**, Phylogenetic tree of 582 rickettsial genomes obtained in this study and 149 rickettsial genomes deposited in the Reference Sequence Database. Different colours represent different groups of *Rickettsia*. TRG, transitional group; TG, typhus group; AG, ancestral group. The genomes assembled in this study are represented by solid dots. **b**,**c**, The phylogenetic tree of *Anaplasma* (**b**) and *Ehrlichia* (**c**), where the size of the dots indicates the number of samples. Red dots indicate a bootstrap value of 100. Reference genomes are represented by grey dots, genomes assembled in this study are marked with turquoise dots, and previously undescribed species identified in this study are annotated in orange. **d**, Orthologous genes among *Ehrlichia*, *Rickettsia* and *Anaplasma*. Red nodes on the phylogenetic tree indicate genomes assembled in this study. In the heat map, orange denotes the presence of orthologous genes in the genome. The numbers in parentheses indicate the count of orthologous genes. **e**, Distribution of endosymbionts across different tick species. The thickness of the chord represents the number of positive samples. Different colours represent different genera of ticks. **f**, Evolutionary relationships of *Coxiella* MAGs assembled in this study, showcasing the identity (colour) and coverage (size) of virulence and nutritional genes, along with genomic collinearity. The calculation method for coverage is the length of the consistent region divided by the full length of the reference gene. The species names are abbreviated as follows: CEAN, *Coxiella* endosymbiont of *Amblyomma nuttalli*; CEOA, *Coxiella* endosymbiont of *Ornithodoros amblus*; CERM, *Coxiella* endosymbiont of *R. microplus*. CLE-Hy, *Coxiella*-like endosymbiont of *Haemaphysalis hystricis*; CLE-Hf, *Coxiella*-like endosymbiont of *Haemaphysalis flava*.

We constructed a phylogenetic tree using the 582 rickettsial genomes obtained in this study, along with all 149 rickettsial genomes deposited in the Reference Sequence Database. The MAGs in this study belong to three of the four known Rickettsiae groups: the spotted fever group (SFG), the transitional group and the typhus group. For the MAGs in the SFG, most of them were classified into one species, yet the phylogenetic tree reveals high genetic diversity within SFG (Fig. 4a). Different tick species tended to carry different *Rickettsia* species or subspecies: *Dermacentor silvarum*, *Rhipicephalus microplus* and *Rhipicephalus turanicus* carried SFG *Rickettsia*, *Ixodes persulcatus* carried ancestral group *Rickettsia*, and *Haemaphysalis* *montgomeryi* primarily carried typhus group *Rickettsia* (Extended Data Fig. 4b). In addition to assembling genomes of four known *Anaplasma* species, we obtained genomes for seven previously undescribed *Anaplasma* species (Extended Data Fig. 4a). Phylogenetic analysis showed that three undefined species formed into a distinct branch closely related to *Anaplasma* *phagocytophilum* (Fig. 4b), a notorious human and animal pathogen. For *Ehrlichia*, we assembled genomes of two known species (*Ehrlichia minasensis* and *Ehrlichia muris*) and six unknown *Ehrlichia* species. The three previously uncharacterized species named *Candidatus* Ehrlichia granulatus, *Candidatus* Ehrlichia microplus-1 and *Candidatus* Ehrlichia javanensis were genetically close to *Ehrlichia* sp. HF, *Ehrlichia canis* and *Ehrlichia ruminantium*, respectively (Fig. 4c). The discovery of these previously undescribed species expands the genomic resources of tick-borne agents in the family Anaplasmataceae and provides insights for early warning of tick-borne pathogens.

To compare genomic signatures among *Rickettsia*, *Anaplasma* and *Ehrlichia*, we identified orthologous genes based on protein similarity. It is worth noting that *Rickettsia* possesses a significantly higher number of unique genes compared to *Anaplasma* and *Ehrlichia* (Fig. 4d). *Rickettsia*-specific genes were particularly enriched in the prokaryotic defence system pathway (Extended Data Fig. 4c), primarily associated with the Type I R-M system. Further Clusters of Orthologous Groups annotation of genus-specific genes indicated that, compared to *Anaplasma* and *Ehrlichia*, *Rickettsia* harbours more genes involved in cellular processes and signalling (Extended Data Fig. 4d). Moreover, we assembled genomes of three *Borrelia* species and investigated the distribution of tick-associated bacteria across different tick species (Extended Data Fig. 4e,f).

Given that ticks are obligate blood-feeding arthropods reliant on vitamin supplementation from endosymbionts^33,35^, we screened clusters to identify *Coxiella*-LE, *Francisella*-LE, *Arsenophonus* and *Candidatus* Midichloria. *Coxiella*-LE was frequently found in *Haemaphysalis*, *Dermacentor*, *Ixodes* and certain soft ticks, albeit with varying prevalence rates (Fig. 4e). *Francisella*-LE was nearly ubiquitous across *Amblyomma* and *Hyalomma* species and occasionally detected in *Haemaphysalis longicornis* ticks (Supplementary Table 8). In addition, phylogenetic analysis of *Rickettsia* endosymbionts identified them in two *Argas persicus* and seven *Amblyomma javanense* (Extended Data Fig. 4g).

*Coxiella*-LE genomes clustered into three distinct clades, including one with *Coxiella* *burnetii*. The phylogenetic position of *Coxiella*-LE showed no clear correlation with tick species or genera (Fig. 4f). *Rickettsia* endosymbionts showed deletions in certain virulence genes (Extended Data Fig. 4h and Supplementary Note 5). *Francisella*-LE genomes clustered into a single phylogenetic clade (Extended Data Fig. 4I). It is worth noting that pathogenicity island genes were either pseudogenized or absent in the *Francisella*-LE genomes analysed.

### Tick–pathogen–microbiome interactions based on hologenome-wide analysis

**Fig. 5:**
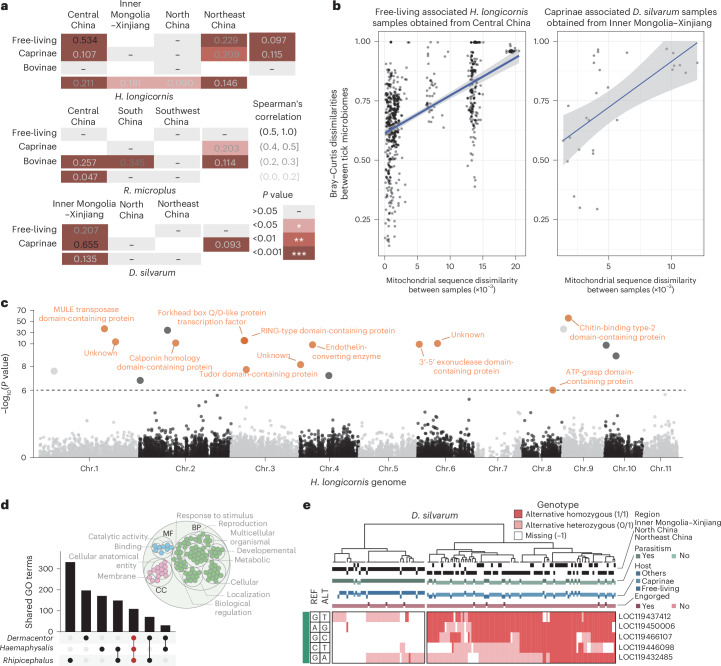
Host genetic variation correlated with pathogen profile. **a**, Spearman correlation between mitochondrial sequence divergence and microbiome community differences in ticks with samples over 200 including *H. longicornis*, *R. microplus* and *D. silvarum* across different sampling regions and parasitic states. The numbers within each heat map cell represent Spearman’s correlation coefficients (at least 20 pairs), and the colour intensity of the heat map reflects the statistical significance of the correlation. **b**, The relationship between mitochondrial sequence divergence and the similarity of tick symbiotic communities for *H. longicornis* in a free-living state from Central China (left) and *D. silvarum* parasitizing sheep from Inner Mongolia–Xinjiang regions (right). Data are presented as fitted values ± 95% confidence interval. **c**, *H. longicornis* genetic variation correlated with the relative abundance of *Rickettsia*. The Manhattan plot shows the results of the mGWAS. Two-sided *P* values were calculated using the BLINK algorithm implemented in the GAPIT R package. Bonferroni correction was applied for multiple comparisons. −log_10_(*P* values) are plotted against the position of SNPs on chromosomes. The gene annotation information corresponding to these SNPs above the threshold is labelled. Chr., chromosome number. **d**, The Upset plot identifies 109 shared GO terms annotated to pathogen-associated genes across three tick species. A total of 119 GO functional annotations are shared among the genes with significant SNPs in the three tick species. MF, molecular function; BP, biological process; CC, cellular component. **e**, Clustering results of samples based on homozygosity of SNPs and the corresponding region, animal host, parasitized state and engorgement condition. Determined to be two. *D. silvarum* clusters were significantly associated with sampling locations (*P* = 0.001, Mantel test), parasitic hosts (*P* = 0.001, Mantel test) and parasitic statuses (*P* = 0.001, Mantel test). REF indicates the nucleotide in the reference genome, whereas ALT represents the alternative nucleotide differing from the reference sequence.

Finally, we have established a comprehensive resource for investigating genetic–microbiome associations in ticks. Correlation analysis revealed significant correlations between mitochondrial genome diversity and microbial community similarity, particularly in free-living *H. longicornis* ticks from Central China and *D. silvarum* ticks parasitizing sheep in Inner Mongolia–Xinjiang (Fig. 5a,b).

These findings sparked our curiosity in exploring the relationship between genetic polymorphisms and pathogen abundance in ticks. We selected three tick species with sufficient sample sizes for analysis: *H. longicornis* ticks (359), *R. microplus* ticks (203) and *D. silvarum* ticks (158). A total of 71,729 high-confidence single-nucleotide polymorphisms (SNPs) were detected in *H. longicornis*, 20,536 in *R. microplus* and 55,530 in *D. silvarum* ticks. We conducted correlation analyses between identified SNPs and pathogen abundance using Bayesian-information and Linkage-disequilibrium Iteratively Nested Keyway (BLINK). This analysis revealed 19 SNPs significantly associated with pathogens in *H. longicornis* and *R. microplus* and 31 in *D. silvarum* (*P* < 10^−6^) (Supplementary Table 9).

**Extended Data Fig. 5:**
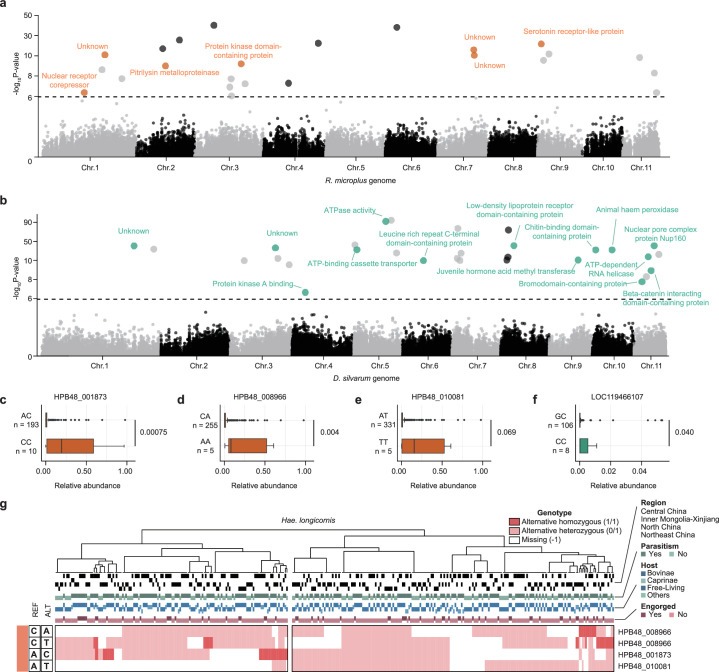
mGWAS of pathogen traits linked with environmental factor. (**a**) *R. microplus* genetic variation correlated with the relative abundance of *Rickettsia*. The Manhattan plot displays the results of the microbiome genome-wide association analysis. -log_10_(P-values) are plotted against the position of SNPs on 11 chromosomes. The significance threshold (dashed line) is set to 1×10^−6^. SNPs above the threshold are shown as large dots. The color of the large dots represents SNPs annotated by SnpEff with high impact or causing missense mutations. (**b**) *D. silvarum* genetic variation correlated with the relative abundance of *Anaplasma*. (**c**-**f**) Relationships between the SNPs homozygosity and the pathogen abundance in different genes of ticks. The pathogen load carried by homozygous individuals of tick SNPs is significantly higher than that in heterozygous individuals. A two-sided Wilcoxon rank-sum test was used for statistical analysis, and no adjustment was made for multiple comparisons. Box plots show the median (center line), the 25th and 75th percentiles (box limits), and whiskers extending to values within 1.5× interquartile range. Outliers beyond this range are shown as individual points. In the C-E, the SNPs originate from the *Hae. longicornis*. The SNPs in the F come from *D. silvarum*. (**g**) Clustering results of *Hae. longicornis* samples based on homozygosity of SNPs.

After assessing the potential functional impact of nucleotide mutations, we identified a SNP on chromosome 4 within a gene encoding a peptidase M13 family protein in *H. longicornis*. This SNP may enhance the *H. longicornis* tick’s blood feeding capabilities. Another SNP, located on chromosome 3 of *H. longicornis* genome within a gene encoding RING-type domain-containing protein, is involved in ubiquitination processes (Fig. 5c). In *R. microplus*, a SNP was identified in the serotonin receptor-like protein gene, which is implicated in the 5-hydroxytryptamine signalling pathway (Extended Data Fig. 5a). In *D. silvarum*, a SNP was discovered in a gene related to juvenile hormone acid methyl transferase (Extended Data Fig. 5b).

To determine whether SNPs shared functional similarities across species, we investigated whether SNPs shared similar functions across species (Supplementary Note 6). Gene Ontology (GO) annotation identified 109 shared GO terms among the three species, primarily related to biological processes such as development, metabolism, localization, cellular processes, reproduction and stress responses (Fig. 5d). A notable relationship emerged between SNP homozygosity and pathogen abundance, with homozygous ticks generally exhibiting higher pathogen loads (Extended Data Fig. 5c–f). Clustering based on SNP homozygosity distinguished distinct sample groups (Fig. 5e and Extended Data Fig. 5g). In *H. longicornis*, cluster formation showed no significant correlation with sampling locations or parasitic status (Extended Data Fig. 5g), whereas in *D. silvarum*, clusters were significantly associated with sampling locations (*P* = 0.001, Mantel test), parasitic hosts (*P* = 0.001, Mantel test) and parasitic statuses (*P* = 0.001, Mantel test). These findings suggest that regional and environmental factors influence genotypic clustering in *D. silvarum*, potentially affecting their microbial community variations.

## Discussion

In this study, we constructed a comprehensive metagenomic database encompassing over 16,000 adult ticks from 48 species across eight genera, representing diverse ecological habitats, geographic regions, and blood-feeding statuses, containing 46 tick species whose microbiome has never been profiled based on metagenomic sequencing. The expanded sampling of dominant tick species enables us to uncover the associations between microbiota composition and ecogeographical factors, host associations and key biological traits of ticks.

Approximately two-thirds of the genomes in our database belong to previously uncharacterized bacterial species, underscoring the vast and unexpected diversity of tick microbiomes. Previous studies on tick microbiome have used the amplicon-based approach targeting the highly conserved 16S ribosomal RNA region^36^ but were insufficient for detecting undefined bacteria. In our previous work, we conducted an initial survey of potential pathogens in ticks from China, offering deeper insights into their vector capacities^23^. In this study, we leveraged metagenomic sequencing and de novo assembly, enabling the identification of both known and unknown bacterial species and offering a more comprehensive perspective on tick microbiomes.

In many cases, human infections are only recognized years after the initial discovery of such potential pathogens, partly due to the markedly lower microbial loads in human blood compared to ticks^37^. For example, *Rickettsia* *sibirica* subspecies *sibirica* BJ-90 was initially detected in *Dermacentor sinicus* ticks from China in 1990 (ref. ^38^), yet it was not confirmed as a human pathogen until 22 years later^39^. Similarly, the 19 previously undescribed tick-associated bacterial species in this study represent potential human pathogens, underscoring the urgent need for enhanced surveillance to enable early identification and prevention of emerging tick-borne diseases.

In *H. longicornis*, two loci showed significant genotype-dependent regulation, with homozygous individuals demonstrating markedly higher *Rickettsia* loads compared to heterozygous counterparts (Extended Data Fig. 5c,d). The SNP on chromosome 3 may influence the ubiquitination pathway by modulating the expression or enzymatic activity of the RING-type ubiquitin ligase gene^40^, thereby regulating tick immune responses. This mechanism potentially alters *Rickettsia*’s capacity to evade the tick immune system, ultimately affecting pathogen load. Studies have demonstrated that ubiquitin ligases restrict the colonization of the rickettsial bacterium *A.* *phagocytophilum* in *Ixodes* *scapularis*, and their silencing confers a survival advantage to this rickettsial bacterium^41^. The mutation in the peptidase family M13 gene on chromosome 4 likely regulates tick vascular function^42^, potentially inducing vascular permeability abnormalities that disrupt tissue microenvironment homeostasis, consequently impacting *Rickettsia* colonization efficiency within ticks. In *R. microplus*, genetic variations in the 5-HT receptor gene showed a significant correlation with *Rickettsia* load. Previous studies demonstrate that serotonin biosynthesis regulates blood-feeding efficiency in ticks^43^, suggesting that 5-HT-mediated neural signalling may modulate pathogen load through critical phenotypes like haematophagy. The JHAMT gene mutation in *D. silvarum* likely regulates *Anaplasma* transmission efficiency by affecting juvenile hormone biosynthesis and subsequent developmental processes^44^.

Overall, we establish a comprehensive large-scale bacterial genome database in ticks, demonstrate the ecogeographical impact on microbiome composition, identify a diverse array of tick-associated bacterial species with pathogenic potential and uncover notable associations between tick genomic diversity and pathogen abundance. Our investigation into the complex interactions among ticks, microbiomes and pathogens enhances our understanding of tick-borne diseases and provides valuable insights for developing targeted tick control strategies.

## Methods

### Sample collection

Ticks were collected from 31 provinces, metropolises or autonomous regions of mainland China. The collection sites were selected according to their ecological environments, including coniferous forest, steppe, farmland, desert, shrubland and tropical forest. Ticks were collected by dragging a standard 1 m^2^ flannel flag over vegetation or from domestic or wild animals such as cattle, dogs, sheep, goats, cats, rabbits, camels, deer and boars. Domestic animals were sampled from local farms, while wild animals were sourced from two origins: wildlife rescue centres and licensed breeding facilities. Ticks were collected with forceps from the animals after obtaining consent from the responsible personnel or owners. The latitude and longitude of each collection site were recorded. Environment factor information was downloaded from the National Geomatics Center of China (https://www.ngcc.cn/). The sample collection includes both free-living and host-attached ticks, encompassing both females and males across a broad geographic range and representing a majority of tick species, ensuring the study’s sample representativeness. The identification of tick species, sex and developmental stage was conducted by an entomologist (Y.S.) based on morphological characteristics, such as the capitulum, palps, scutum, coxae and tarsi, following the genus and species identification key^45^. Live ticks were transported to the laboratory, and dead ticks were directly stored at −80 °C.

### Metagenomic sequencing

Adult ticks were used for tick metagenomic sequencing. Tick samples were pooled according to species, sex, collection site and blood-feeding status, and the total DNA was extracted from each pool. Each individual tick was exclusively used for either Illumina or nanopore sequencing. All 1,460 adult tick samples collected from the wild were thoroughly surface sterilized (two successive washes of 70% ethanol, 30 s each), and genomic DNA for resequencing was isolated using the AllPrep DNA/RNA Mini Kit (QIAGEN, catalogue number 69504). The DNA concentration was measured using the Qubit dsDNA HS Assay Kit in a Qubit 2.0 fluorometer (Life Technologies). Sequencing libraries were constructed using the NEBNext UltraTM DNA Library Prep Kit for Illumina (NEB) following the manufacturer’s recommendations, and index barcodes were added to attribute sequences to each sample. The library preparations were sequenced on an Illumina NovaSeq platform (NovaSeq 6000 SP Reagent Kit). Each sample generated approximately 70 million paired-end reads (150 bp × 2), resulting in about 20 Gb of sequencing data.

### Nanopore sequencing

Live adult ticks were used for tick nanopore sequencing. The individuals used for nanopore sequencing were independent of those used for Illumina sequencing. Each pooled sample, comprising a single tick species, contained approximately 15–30 adult ticks. High-quality DNA extraction is initially conducted using Qiagen Genomic Kit (catalogue number 13443). Subsequently, a comprehensive assessment of the DNA sample’s suitability is performed through a multi-faceted approach. This includes a visual inspection for potential contaminants or irregularities in the sample’s appearance. In addition, a 0.75% agarose gel electrophoresis is used to ascertain any degradation and determine the size of DNA fragments present. The assessment further involves the use of Nanodrop to evaluate DNA purity, ensuring an optimal optical density at 260/280 nm (OD260/280) ratio between 1.8 and 2.0 and an optical density at 260/230 nm (OD260/230) ratio falling within the range of 2.0 to 2.2. Finally, Qubit is used for precise quantification, ensuring that the DNA meets the required standards for concentration and quality. After the quality assessment of the DNA sample, BluePippin automated nucleic acid size selection system is used to recover specific fragment sizes of DNA. Subsequently, the DNA fragments obtained after size selection undergo damage repair and end repair procedures. Following magnetic bead purification, a DNA barcode is ligated to the DNA ends using a kit. After another round of magnetic bead purification, sequencing adapters provided within the ligation kit are ligated to the DNA. Finally, the constructed DNA library undergoes precise quantification using Qubit to ensure accurate quantitation of the library. After constructing the library, a specific concentration and volume of the DNA library is loaded onto a flow cell. The flow cell is then transferred to the PromethION sequencer for real-time single-molecule sequencing.

### Genome assembly and genome annotation for nanopore sequencing data

We used Porechop v0.2.4 (ref. ^46^) to trim chimeras and remove sequence adapters for raw nanopore sequencing data. Nanofilt v2.6.0 (ref. ^47^) was used to filter out low-quality and short reads, while Filtlong v0.2.0 (https://github.com/rrwick/Filtlong) was applied to eliminate Nanopore sequences of poor quality and shorter than 1,000 bp, retaining 95% of the data. The clean data were used to assemble by Flye v2.8 (ref. ^48^) for continuity with a previous study (ref. ^49^). Racon v1.4.13 (ref. ^50^) was used for correction by integrating nanopore sequencing data with one round of polishing (Supplementary Note 7). After extracting DNA for nanopore sequencing, a portion of the DNA was also subjected to Illumina sequencing workflow to obtain reads for Illumina-reads-based genome polishing by Pilon v1.23 (ref. ^51^) and NextPolish v1.3.0 (ref. ^52^). To further improve the completeness of the genome assembly, we performed de novo assembly of the Illumina read data using MEGAHIT v1.2.9 (ref. ^53^) and subsequently integrated the corrected Nanopore-read assembly with the Illumina-read assembly using QuickMerge v0.3 (ref. ^54^). Kraken2 v2.1.0 (ref. ^55^) was used to identify the bacterial contigs in assembly. Contigs with fragments annotated as bacterial sequences were filtered out, representing approximately 0.1% of the assembled sequences. The quality of the assembly was assessed using QUAST v5.0.2 (ref. ^56^), and the completeness was evaluated using BUSCO v5.2.2 with arthropoda_odb10 (ref. ^57^). Repetitive sequences in each tick genome were identified based on RepeatModeler v2.0.5 (ref. ^58^). Gene annotation was accomplished by integrating evidence or predictions from transcriptome-, ab initio- and homology- based approaches. In the transcriptome-based approach, RNA-sequencing data for each tick species was acquired from a previous study in the National Center for Biotechnology Information (NCBI) Sequence Read Archive under Bioproject PRJNA841744. RNA-sequencing reads generated from each tick species were assembled by Trinity v2.4.0 (ref. ^59^) with default parameters. The assembled transcripts were aligned to each assembled genome and were used to predict gene structure by PASA v2.3.3 (ref. ^60^). Ab initio gene prediction was performed using Augustus v3.3 (ref. ^61^). Homologous protein sequences were aligned to the tick genome assemblies using TBLASTN v2.2.28+ (ref. ^62^), and Genomethreader v5.4.0 (ref. ^63^) was used to predict gene structure. Finally, we used EvidenceModeler (EVM) v1.1.1 (ref. ^64^) to integrate the gene models predicted by the above approaches into a nonredundant and more complete gene set.

### Phylogenetic analysis and divergence time estimation for tick genome

The single-copy orthologue proteins within *I. persulcatus*, *H. longicornis*, *D. silvarum*, *Hyalomma asiaticum*, *Rhipicephalus sanguineus*, *R. microplus*, *I. scapularis*, *Centruroides sculpturatus* and *Parasteatoda tepidariorum* were identified using orthofinder v2.5.4 (ref. ^65^) with default parameters. A total of 3,150 orthologous protein sequences were identified and considered the core protein. The protein sequences annotated from genomes assembled by nanopore sequencing reads were mapped to core proteins, and aligned protein sequences were extracted for phylogenetic tree construction. Extracted protein sequences were aligned using MUSCLE v3.8.1551 (ref. ^66^). Gblocks v0.91b (ref. ^67^) was used to select conserved blocks from aligned sequences. Finally, iqtree v2.2.0 (ref. ^68^) was used to generate a phylogenetic tree and calculate branch support. The divergence time within the nodes of the phylogenetic tree was estimated by r8s (ref. ^69^).

### Assembly and phylogenetic tree construction for tick mitochondrial genome

Raw Illumina reads were filtered using the AfterQC v0.9.6 (ref. ^70^) with default parameters. MitoZ v3.6 (ref. ^71^) was used to assemble mitochondrial genome, which performs de novo assembly based on all reads and subsequently filters non-mitochondrial contigs and annotates the mitochondrial scaffolds. For tick species with less than 50 samples, all available samples were used for mitochondrial genome assembly; for tick species with more than 50 samples, a random subset of 50 samples was selected. The longest mitochondrial genome sequence obtained for each species was ultimately chosen for downstream analysis. We initially selected the nucleotide sequences of 23 genes, including *ATP6*, *ATP8*, *COX1*, *COX2*, *COX3*, *ND1*, *ND2*, *ND3*, s-rRNA, *trnA*, *trnC*, *trnD*, *trnE*, *trnG*, *trnK*, *trnL*, *trnM*, *trnN*, *trnR*, *trnS*, *trnV*, *trnW* and *trnY*. For each gene listed, protein sequences were taken from all mitochondrial assemblies and subjected to multiple sequence alignment using MUSCLE v3.8.1551 (ref. ^66^). Gblocks v0.91b (ref. ^67^) was used to select conserved blocks for each alignment. Finally, iqtree v2.2.0 (ref. ^68^) was used to generate a phylogenetic tree and calculate branch support. Mitochondrial dissimilarity was extracted from the phylogenetic tree. The geographical distance between two samples was calculated as follows: Geographical distance = cos^−1^(cos(*E*_1_ − *E*_2_) × cos(*N*_1_) × cos(*N*_2_) + sin(*N*_1_) × sin(*N*_2_)) × 6,371 km, where *E*_1_ and *N*_1_ were the longitude and latitude of sample 1, and *E*_2_ and *N*_2_ were the longitude and latitude of sample 2.

### Genome assembly and binning for metagenomic sequencing data

Illumina reads after quality control were aligned to the tick genomes required above using the bowtie2 v2.4.1 (ref. ^72^) with default parameters. The paired reads were discarded if one read matched the tick genome by using samtools v1.9 (ref. ^73^) with parameters -f 12. For samples with remaining sequence data greater than 10 G, we used MEGAHIT v1.2.9 (ref. ^53^) for assembly. For samples with less than 10 G of remaining sequence data, SPAdes v3.15.2 with --meta option^74^ was used for assembly. A test of this selection strategy was provided in Supplementary Note 8. Binning and genome reconstruction were accomplished by MetaBAT2 v2.2.15 (ref. ^75^). Assembly quality was assessed through CheckM v1.1.3 in lineage_wf workflow^76^, and MAGs meeting criteria of at least 50% completeness and less than 10% contamination as Genomic Standards Consortium recommended were used for downstream analysis. Prokka v1.14.6 (ref. ^77^) was used to identify the genes with default parameters.

### MAGs clustering and phylogenetic tree construction

ANI among each genome was calculated by fastANI v1.32 (ref. ^29^). Louvain v0.16 (ref. ^78^) on Python 3.10.0 was used to cluster and identify the groups of MAGs. We assessed and scored the genomes based on factors such as genome completeness, contamination ratio, the number of contigs, circularity, presence of the 16S gene and the count of transfer RNA genes. The genome with the highest quality was selected as the representative genome. PhyloPhlAn v3.0.60 (ref. ^79^) was used to construct a microbial phylogenetic tree.

### Taxonomically profiling and ecotype analysis

After host sequences were filtered out, species classification was performed using Kraken2 v2.1.0 (ref. ^55^) to determine the abundance of microbes in each sample. Based on the microbial communities at the genus level, samples were classified into five ecotypes. Hierarchical clustering (Ward D2 method) is performed based on the taxonomic profile of the microbial community at the genus level. The optimal number of clusters was determined using the elbow method. This method involves selecting the point where the change in intra-cluster distance is minimized as the cluster count is varied. This point indicates that beyond it, the trend of distance change begins to plateau, suggesting that further increases in the number of clusters might not substantially enhance the accuracy or interpretability of the clustering. Representative samples for each ecotype are defined as follows: First, low-dimensional embeddings of the samples are calculated based on isometric mapping. For each ecotype ET_*i*_, the sample that is furthest from the centre of all samples is considered the most representative and is denoted as *S*_*i*,rep_. The distance from this representative sample to the centre of all samples is recorded as *D*_*i*,rep_. For each ecotype ET_*i*_, we calculate the distance from every sample *S*_*i*,*j*_ belonging to this ecotype to *S*_*i*,rep_, denoted as *D*_*i*,*j*_. If *D*_*i*,*j*_ is less than 0.5 × *D*_*i*,rep_, we consider it a representative sample. Vegan v2.6-2 (ref. ^80^) package in R was used to calculate the Shannon index, and dimensionality reduction was performed with phateR (ref. ^81^). To investigate the effects of ticks, parasitizes, habitats and environmental factors on the tick-associated microbiomes, vegan v2.6-2 (ref. ^80^) in R was used to calculate Bray–Curtis distances between samples. Dimensionality reduction was conducted using Rtsne (ref. ^82^) in R, and differences in distances between groups of different ticks, hosts, habitats and environmental factors were compared using the adonis function for PERMANOVA^32^ on datasets with sample sizes exceeding 8 to analyse the impact of these factors on the microbiota. Significance of environmental influences on tick microbiome was calculated using A3 package, and only influencing factors with a significance level below 0.05 were displayed.

### Potentially pathogenic bacteria identification and species classification

PhyloPhlAn v3.0.60 (ref. ^79^) was used to construct a microbial phylogenetic tree. For each genome, Kraken2 v2.1.0 (ref. ^55^) was used for taxonomy annotation, and genomes annotated as *Rickettsia*, *Anaplasma*, *Ehrlichia*, *Coxiella*, *Francisella*, *Borrelia*, *Candidatus* Midichloria and *Arsenophonus* were selected as tick-associated bacteria. The Core Genome Alignment Sequence Identity were estimated for each cluster to classify them into species. The Core Genome Alignment Sequence Identity ≥96.8% among genomes were considered indicative of the same species, in accordance with the criteria used especially for the order Rickettsiales^83^. Orthologous genes were identified using orthofinder v2.5.4 (ref. ^65^). *Anaplasma* and *Ehrlichia* showed a greater number of unique orthologous gene clusters compared to *Rickettsia*, which shared fewer orthologues with the other two genera. Clusters of Orthologous Groups annotation was accomplished by emapper v2.1.9 (ref. ^84^) based on eggNOG DB v5.0.2 (ref. ^85^). Virulence and vitamin biosynthesis protein in genomes were identified using diamond v0.9.35 (ref. ^86^) based on these proteins in the virulence factor database^87^ and NCBI.

### SNP identification

Owing to their considerable sample sizes and substantial research importance of *H. longicornis*, *R. microplus* and *D. silvarum*, the samples from these three tick species were used for microbiome genome-wide association study (mGWAS) analysis. Reference genome and their corresponding genome annotation files (GFF) for the *H. longicornis*, *R. microplus* and *D. silvarum* were downloaded from the NCBI Genome database. BEDTools v2.27.1 (ref. ^88^) was used to extract the exonic sequences from the reference genome sequence based on GFF files, and SeqKit v2.3.0 (ref. ^89^) was used to deduplicate the sequences. Illumina reads after quality control were aligned to the reference genome exonic sequences using Bowtie2 v2.4.2 (ref. ^72^), and the generated SAM files were processed by SAMtools v1.9 (ref. ^73^) to generate BAM files and indices. Variant identification was performed using GATK4 v4.0.12.0 (ref. ^90^). The Genome Analysis Toolkit (GATK) MarkDuplicates was used to mark duplicated reads caused by PCR and cloning. Alignment summary metrics, such as alignment rate, were summarized by GATK CollectAlignmentSummaryMetrics. The deduplicated BAM files were subjected to variant calling using GATK HaplotypeCaller, and GVCF files were generated. GVCF files were converted to VCF format using GATK GenotypeGVCFs. VCF files were compressed, indexed and merged using BCFtools v0.1.16 (ref. ^73^) to produce a consolidated VCF file for each tick species. SNPs were filtered and marked using GATK VariantFiltration based on quality control (quality by depth (QD) < 2.0, quality score (QUAL) < 30.0, Fisher strand bias (FS) > 60.0, mapping quality (MQ) < 40.0, mapping quality rank sum (MQRankSum) < −12.5 and read position rank sum (ReadPosRankSum) < −8.0). GATK SelectVariants was then used to exclude the marked low-quality SNP from the VCF file. Further filtering of SNPs was performed using vcftools^91^ according to the criteria of a missing rate less than 50% (--max-missing 0.5) and a minor allele frequency greater than 5% (--maf 0.05). The filtered result files were sorted using TASSEL v5.2.40 (ref. ^92^) and converted from VCF format to HapMap format. After quality control, 20,547 SNPs were retained for *H. longicornis*, 55,541 for *R. microplus* and 71,740 for *D. silvarum*.

### Microbiome GWAS analysis

To ensure more reliable results, we set thresholds based on a relative abundance greater than 0.001 in each individual sample and a prevalence greater than 15% in the respective tick species for pathogen selection. Ultimately, the candidate associated pathogens identified were *Rickettsia* for *H. longicornis*, *Rickettsia* for *R. microplus* and *Rickettsia* and *Anaplasma* for *D. silvarum*. Association analysis between genotypes and phenotypes was conducted using GAPIT v3.2.0^93^ in R, and BLINK^94^ algorithm from the mixed linear model was used to conduct association detection. The significance threshold (*P* value cut-off is set to 1 × 10^−6^) was determined by the Bonferroni test. After completing the association analysis, SNPs located on chromosomes were retained for subsequent bioinformatics analysis. We used the emapper v2.1.9^84^ software to functionally annotate genes identified with SNPs. In addition, we used the genetic variant annotation software SnpEff v5.2^95^ to analyse the functional impact of SNPs obtained from mGWAS analysis, predicting the effects of these SNPs on the function of the encoded proteins in the respective genes. PLINK v1.90b4^96^ was used to perform linkage disequilibrium analysis on SNPs. Independent genetic variant loci were selected using thresholds of LD *r*^2^ < 0.1 and *P* < 10^−^^6^. GAPIT^93^ was used to evaluate the contribution of these independent variant loci to the explanation of phenotypic variance. We performed hierarchical clustering on tick samples based on the genotypes of selected SNPs and evaluated the correlation between SNP genotypes and tick sample metadata using the Mantel test. Samples were further grouped according to genotype, and SNPs with a larger number of samples were selected for subsequent analysis. The Wilcoxon rank-sum test was used to assess the relationship between genotypes and the abundance of corresponding pathogens. All statistical analyses were conducted using R software, with *P* < 0.05 indicating statistical significance. Hierarchical clustering (Ward D2 method) was performed based on the SNP alleles after the genotype data were converted to character variables. During the clustering process, distance matrices between samples or SNPs are computed, and hierarchical clustering is conducted based on these distances. Silhouette analysis is used to select the optimal number of clusters.

### Reporting summary

Further information on research design is available in the Nature Portfolio Reporting Summary linked to this article.

## Supplementary information

Supplementary Information Supplementary Figs. 1–4, Notes 1–8, Discussion and References.

Reporting Summary

Supplementary Tables Supplementary Tables 1–13.

## Data Availability

The genome assemblies for 19 tick species in this study are available via European Nucleotide Archive (ENA) (PRJEB89832) at https://www.ebi.ac.uk/ena/browser/view/PRJEB89832 and via the National Genomics Data Center (NGDC) (PRJCA037692) at https://ngdc.cncb.ac.cn/bioproject/browse/PRJCA037692. The raw Illumina sequencing data are available via ENA (PRJEB89768) at https://www.ebi.ac.uk/ena/browser/view/PRJEB94929, and via NGDC (PRJCA017096) at https://ngdc.cncb.ac.cn/bioproject/browse/PRJCA017096 and (PRJCA002242) at https://ngdc.cncb.ac.cn/bioproject/browse/PRJCA002242. The genomes for 1,373 rMAGs in this study are available via ENA (PRJEB89768) at https://www.ebi.ac.uk/ena/browser/view/PRJEB89768 and via NGDC (PRJCA037694) at https://ngdc.cncb.ac.cn/bioproject/browse/PRJCA037694.

## Code availability

The code used for the analysis is available via Zenodo at 10.5281/zenodo.16248308 (ref. ^97^).
