# Supplementary material for: Genome-resolved metagenomics reveals microbiome diversity across 48 tick species
Source: Nat Microbiol. 2025 Sep 23;10(10):2631–45. doi: 10.1038/s41564-025-02119-z (PMC12488481; doi:10.1038/s41564-025-02119-z)
Supplement: Supplementary file 1 — Supplementary Figs. 1–4, Notes 1–8, Discussion and References. [file 41564_2025_2119_MOESM1_ESM.pdf]

nature microbiology

Supplementary information

https://doi.org/10.1038/s41564-025-02119-z

Genome-resolved metagenomics reveals microbiome diversity across 48 tick species

In the format provided by the authors and unedited

Supplementary Figures

Supplementary Fig. 1

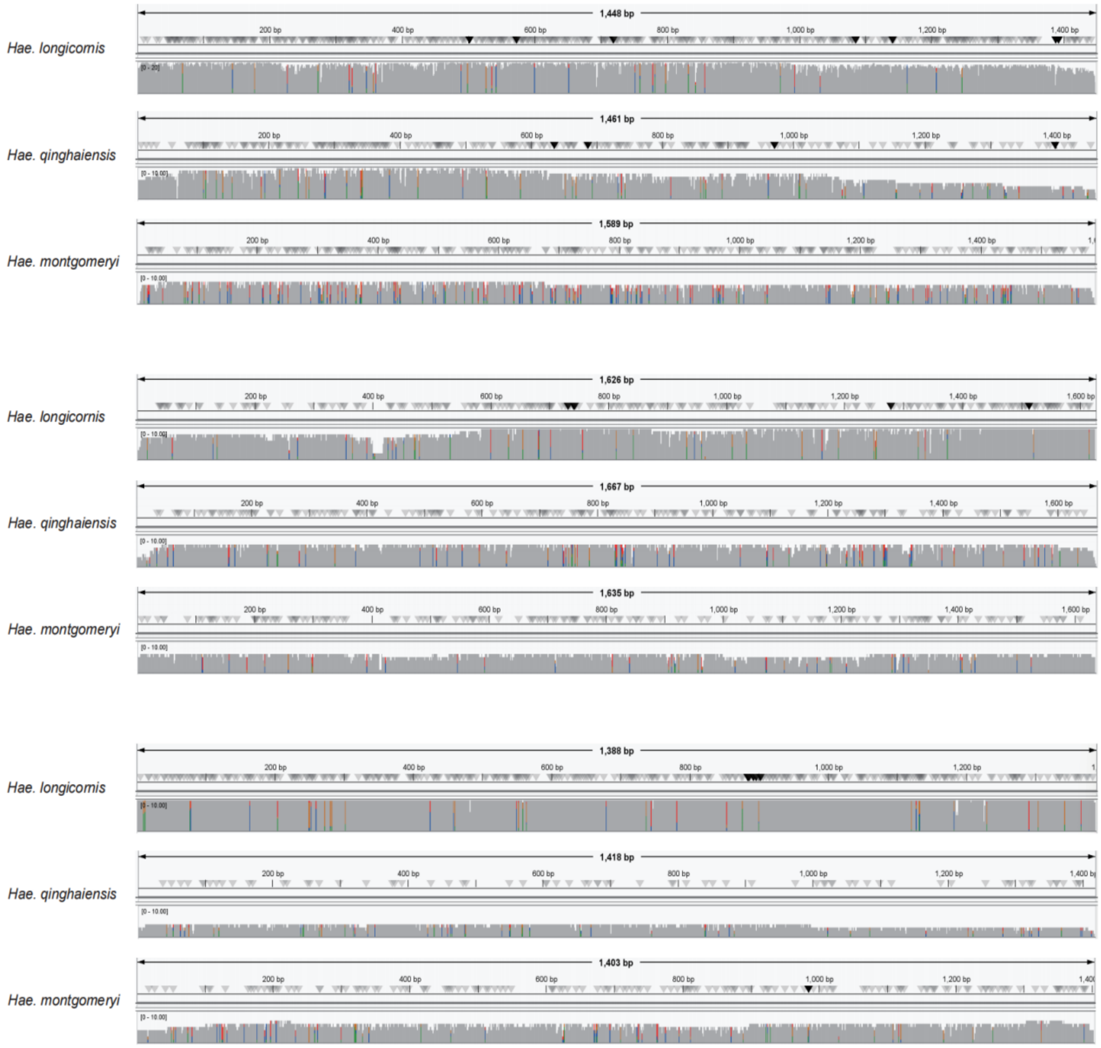

Supplementary Fig. 1. Comparison of variations in homologous regions among *Hae. qinghaiensis* and *Hae. montgomeryi* and their related species *Hae. longicornis*. Long reads were aligned to homologous regions from three genomes using minimap, and the resulting alignments were visualized with IGV.

Supplementary Fig. 2

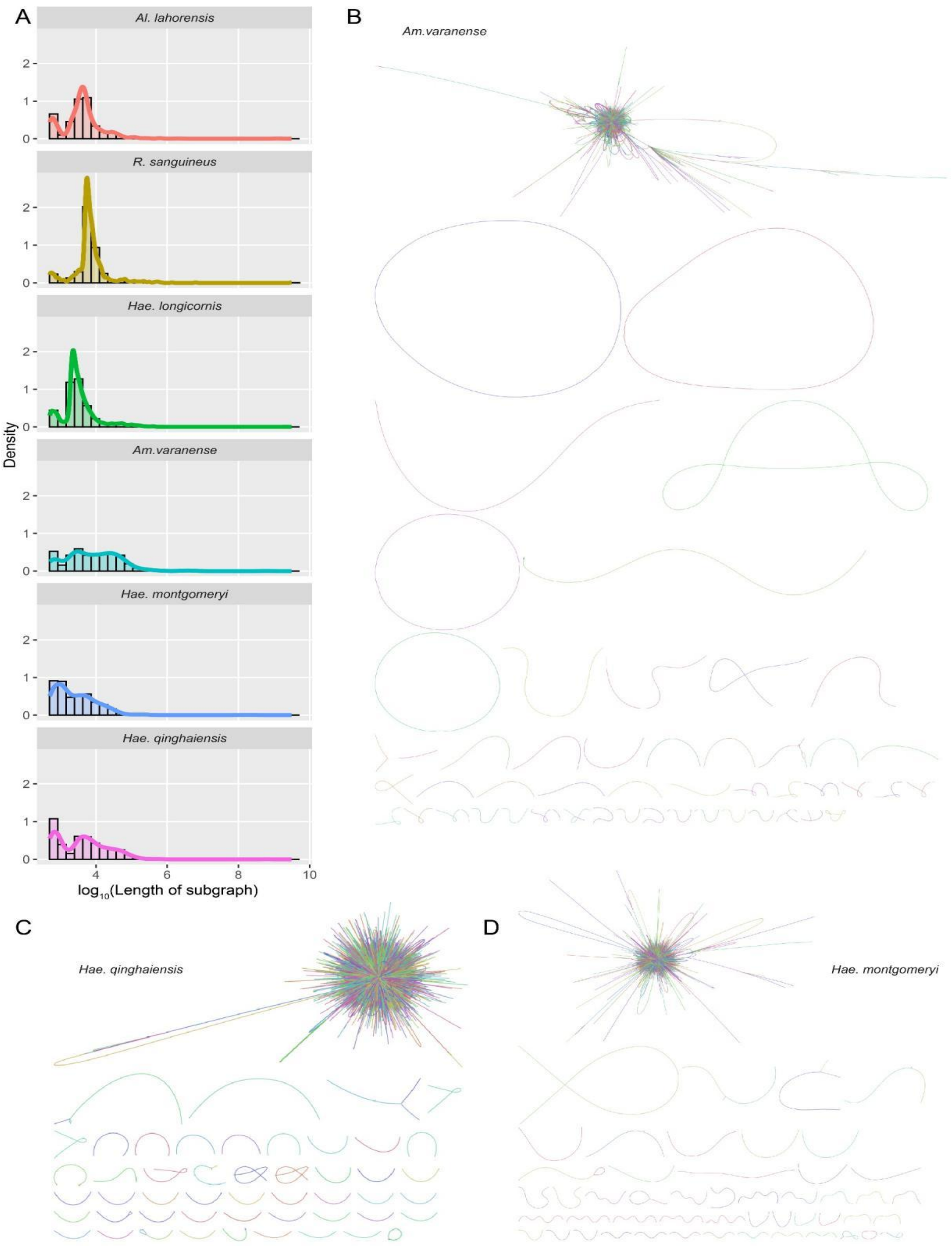

Supplementary Fig. 2. Histogram of subgraph length distribution (A) and the top 50 assembly subgraphs (B-D) of *Am.varanense*, *Hae. montgomeryi* and *Hae. qinghaiensis*. (The assembly subgraphs of the first three samples are too large to visualize).

Supplementary Fig. 3

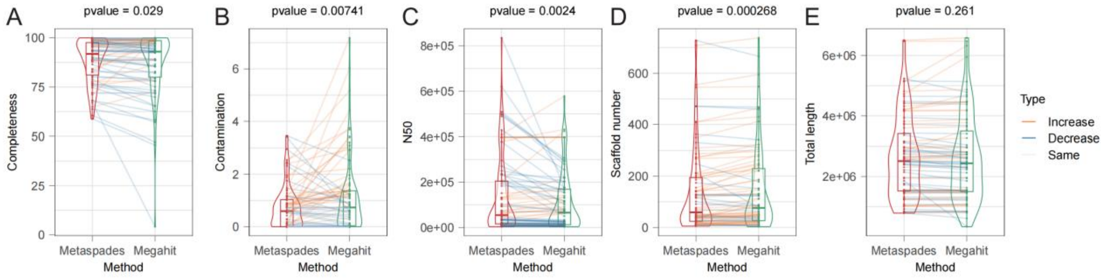

Supplementary Fig. 3. Comparisons of completeness (A), contamination (B), N50 (C), scaffold number (D) and total length (E) of MAGs assembled by MEGAHIT and metaSPAdes. Two sided Wilcoxon signed-rank test was used based on characteristics of 86 MAGs. Box plots show the median (center line), the 25th and 75th percentiles (box limits), and whiskers extending to values within 1.5× interquartile range. Violin plots show the distribution of the data with the width representing the density of data points.

Supplementary Fig. 4

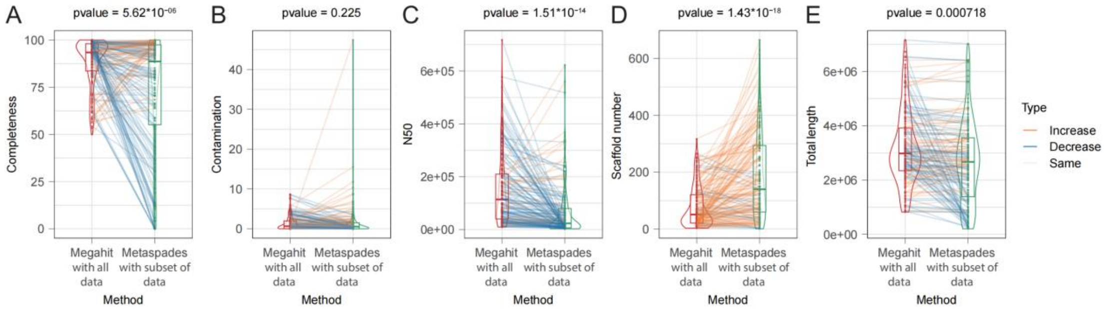

Supplementary Fig. 4. Comparisons of completeness (A), contamination (B), N50 (C), scaffold number (D) and total length (E) of MAGs assembled by MEGAHIT with all data and metaSPAdes with downsampled data. Two sided Wilcoxon signed-rank test was used based on characteristics of 157 MAGs. Box plots show the median (center line), the 25th and 75th percentiles (box limits), and whiskers extending to values within 1.5× interquartile range. Violin plots show the distribution of the data with the width representing the density of data points.

Supplementary Notes

Supplementary Note 1 | Phylogenetic relationship of ticks based on mitochondrial and nuclear genomes

In the samples we collected, each tick genus was overrepresented by one dominant species. For example, 64% of *Haemaphysalis* samples were *Hae. longicornis*, 82% of *Dermacentor* were *D. silvarum*, and 59% of *Rhipicephalus* were *R. microplus*. Phylogenetic analysis based on mitochondrial genomes delineated two distinct clades for hard (ixodid) and soft (argasid) ticks, with the genus *Ixodes* (representing Prostriata) notably diverging from other Metastriata genera. Genera such as *Amblyomma*, *Dermacentor*, *Rhipicephalus*, and *Hyalomma* clustered closely together, exhibiting a more distant evolutionary relationship with the genus *Haemaphysalis*. Phylogenetic analysis based on these draft genomes aligned with previous findings for the six previously sequenced tick species[23]. Moreover, the results closely resembled the mitochondria-based phylogenetic tree (Fig. 1c, 1e), reinforcing the accuracy of our classification and identification processes.

Supplementary Note 2 | The quality of *Hae. qinghaiensis* and *Hae. montgomeryi* genomes

The relatively lower assembly quality observed in these two species is likely due to their lower sequencing depth compared to other species. Specifically, the sequencing data obtained for *Hae. qinghaiensis* and *Hae. montgomeryi* were less than 20 Gb, which is notably lower than the over 30 Gb generated for more complete species such as *Hy. anatolicum* and *Al. lahorensis*.

We detected homologous regions and SNPs in *Hae. qinghaiensis* and *Hae. montgomeryi*, as well as in their related species, *Hae. longicornis*. A comparative analysis of assembled contigs and long-read mapping (with sequencing errors excluded based on the variation cutoff) revealed that the genomes with lower completeness (*Hae. qinghaiensis* and *Hae. montgomeryi*) exhibited a higher proportion of variations compared to the more complete genome of *Hae. longicornis* (Supplementary Fig. 1). This increased genomic diversity may partially explain the

reduced assembly completeness in the two tick species.

We present statistics from the GFA files generated during the assembly of six randomly selected samples, including the number of nodes, branches, and subgraph sizes (Supplementary Table 10). The results indicate that all samples are dominated by a single large subgraph (Supplementary Fig. 2). For *Al. lahorensis* and *R. sanguineus*, the largest subgraph exceeds 10^9 bp, with branch counts ranging from 2×10^4 to 3×10^4. In contrast, for *Hae. montgomeryi* and *Hae. qinghaiensis*, the branch counts are approximately 4×10^4 and 2.4×10^4, respectively, and their largest subgraphs have roughly one-tenth the size. Similarly, while *Hae. longicornis* has a large subgraph, its high branch count results in lower assembly continuity compared to the first two species. These statistics indicate that, for a given graph size, a higher level of genetic variation leads to more branches, which in turn reduces assembly continuity. This may be due to individual differences in mixed tick samples or species-level variations.

Supplementary Note 3 | Clusters of tick samples based on metagenome-assembled genomes (MAGs)

Cluster I consisted of four tick species from three genera primarily found in Central and Southern China. Cluster II contained two tick species from the genera *Hyalomma* and *Rhipicephalus*, clustered in the Inner Mongolia-Xinjiang region. Cluster III included two species from two genera distributed in Yunnan and Hainan provinces. These first three clusters appeared to be primarily shaped by geographical fauna, suggesting that environmental factors play a key role in microbial community composition. Cluster IV consisted of three *Dermacentor* tick species clustered in Northeast and North China. Cluster V included three *Haemaphysalis* tick species widely distributed in the Eastern region (encompassing much of North China, Northeast China and Central China). The high similarity within Clusters IV and V may be attributed to their shared taxonomic lineage, as species within the same genus tend to harbor more similar microbiomes. Cluster VI exhibited lower similarity, featuring *I. persulcatus* from the Northeast China, and *I. ovatus* and *Hae. qinghaiensis* clustered in Qinghai province.

Supplementary Note 4 | Characteristics of five tick microbiome ecotypes

Given the intricate nature of tick microbial communities and the intertwined effects of multiple factors, isolating the independent impacts of each factor present a challenge. Drawing on the concept of "enterotype" used in gut microbiome studies. ET_1 was characterized by *Rickettsia*, ET_2 by *Staphylococcus*, ET_3 by *Pseudomonas*, ET_4 by both *Bacillus* and *Francisella*, while ET_5 displayed greater microbial heterogeneity, dominated by *Acinetobacter*, *Corynebacterium*, *Stenotrophomonas*, and *Serratia*. Factors influencing the microbiome were categorized into three tiers based on their impact: the primary tier included tick species, host, and region; the second tier included factors like mean minimum temperature (Tmin), parasitized status, mean temperature (Tmean), mean relative humidity (RHmean), mean and maximum precipitation (Prmean, Prmax); all other factors were classified in the tertiary tier. These findings suggest that tick microbiomes are primarily shaped by the ticks themselves, followed by animal hosts, geographic distribution, and environmental conditions, a pattern that closely aligns with the hierarchical structure observed in ecological food chains.

Supplementary Note 5 | Genomic characterization of tick-associated bacteria

Two new species, named *Candidatus* Anaplasma microplus and *Candidatus* Anaplasma abaensis, clustered with *A. platys* and *A. ovis*, respectively. The remaining three new species, identified in *Hae. tibetensis*, *Hae. longicornis*, *R. microplus*, formed a unique branch, with biological traits and pathogenic potential warranting additional research. Functional annotation revealed that *Rickettsia*-specific genes are enriched in the lipopolysaccharide-related pathway, potentially linked to the absence of lipopolysaccharide in *Anaplasma* and *Ehrlichia*. The genome sizes of *Coxiella*-LE ranged from 0.6 to 1.6 Mb, significantly smaller than those of *C. burnetii* (~2 Mb). Key virulence genes encoding proteins for the Dot/Icm secretion system were either pseudogenized or entirely absent in *Coxiella*-LE genomes. However, genes involved in B-vitamin synthesis (B2, B5, B6, B7, and B9) were consistently retained, reinforcing their role as nutritional endosymbionts. Genome sizes for *Francisella*-LE ranging from 1.41 to 1.62 Mb, notably smaller than pathogenic *F. tularensis* genomes

(1.79 Mb) and genes responsible for the biosynthesis of B2, B7, and B9 vitamins were well-conserved. For *Rickettsia* endosymbionts, their genome sizes (1.4-1.5 Mb) were larger than those of pathogenic rickettsiae (1.0-1.3 Mb).

Supplementary Note 6 | Tri-interaction of tick-pathogen-microbiome

To minimize the confounding effects of sampling location and parasitic status, Spearman correlation was calculated between mitochondrial sequence dissimilarities and Bray-Curtis dissimilarities, restricting comparisons to samples from the same locations and with identical parasitic statuses. We identified single nucleotide polymorphisms (SNPs) within their genomes and performed correlation analyses between SNPs and pathogen abundance and focused on bacteria with higher abundance in these ticks. Although no common KEGG pathways were identified among the three tick species, *Hae. longicornis* and *R. microplus* share pathways related to the angiotensin converting enzyme, reinforcing its potential significance in tick[98].

Supplementary Note 7 | Tick genome assembly polish

We used Nanopore sequencing data to correct contigs following the method described in the Racon documentation (https://github.com/isovic/racon). After extracting DNA for Nanopore sequencing, a portion of the DNA was also subjected to Illumina sequencing workflow to obtain second-generation sequencing reads for polishing genome. Pilon v1.23 and NextPolish v1.3.0 were used for Illumina-reads-based genome polishing. In the original Racon publication, the authors employed miniasm to generate draft assemblies, which differs from the assembler used in our study. We performed only one round of polishing with Racon, reducing the overall genome size and N50 by approximately 1%-5%. An additional round of correction was performed. However, it did not result in notable improvements in N50 or genome size (Supplementary Table 11).

Supplementary Note 8 | Comparative evaluation of binning results from

assemblies generated by metaSPAdes and MEGAHIT

To assess whether assembly quality was compromised when MEGAHIT was used, we compared its performance to that of metaSPAdes on several smaller datasets. Additionally, we downsampled some of the largest datasets and assembled them using metaSPAdes to evaluate the impact of assembler choice under comparable conditions. To compare the performance of MEGAHIT to metaSPAdes on smaller datasets, we randomly selected 25 samples, each containing approximately 6 million reads, and performed assemblies using MEGAHIT. We then compared the assembly performance between MEGAHIT and metaSPAdes on these smaller datasets. In terms of bin recovery, MEGAHIT successfully identified 86 of the 91 bins obtained by metaSPAdes. Regarding individual bacterial genomes, metaSPAdes outperformed MEGAHIT in terms of scaffold number, completeness, contamination, and N50, with statistically significant differences (P-value < 0.05, Wilcoxon signed-rank test, Supplementary Fig. 3).

To evaluate the performance of metaSPAdes on downsampled versions of the largest datasets, we selected 25 of the largest datasets and used seqkit to sample 6 million paired reads from each, followed by assembly using metaSPAdes. We then compared the bacterial genomes recovered from the sampled assemblies with those obtained by assembling the complete datasets using MEGAHIT. In terms of bin recovery, the metaSPAdes assembly on the downsampled data produced 157 bins, while the full MEGAHIT assembly yielded 438 bins. Moreover, the quality of the assembled genomes, as reflected by completeness, N50, scaffold count, and total length, was significantly affected by the sampling process (P-value < 0.05, Wilcoxon signed-rank test, Supplementary Fig. 4).

Supplementary Discussion

By integrating both short-read and long-read high-throughput sequencing, our hologenomic analysis has significantly enhanced the efficiency of constructing metagenome-assembled genomes, laying a foundation for investigating functional aspects of tick biology[12]. Previous studies primarily used amplicon-based methods

targeting the conserved 16S rRNA region[36], providing genus-level bacterial composition data. However, these methods limit the resolution of bacterial species diversity and obscure the intricate interactions between ticks and their microbiota. Additionally, amplicon sequencing is prone to artificial bias during PCR amplification, often leading to quantification inaccuracies and affecting the assessment of microbial diversity[99]. In contrast, whole-genome shotgun metagenomic sequencing provides an unbiased and enrichment-free approach, allowing for a more comprehensive analysis. Moreover, the generation of high-quality metagenome-assembled genomes has allowed us to perform whole-genome investigations and high-resolution phylogenetics, methodologies that were previously only achievable through the much lower-throughput processes of culturing or genome isolation. With such a sequencing strategy, the paired data of tick genome and microbiome obtained in this study allows us, for the first time, to perform hologenomic analyses of the ticks and their commensal microbes on a large scale for exploring tick-microbiome interaction.

Among the thousands of metagenome-assembled genomes analyzed in this study, we identified 712 tick-borne agents, comprising 13 known species and 19 previously uncharacterized species. Although their pathogenicity remains unclear and requires experimental validation, these microbes could potentially contribute to the emergence of new tick-borne diseases, posing substantial public health risks. Our findings further support the perspective that metagenomic sequencing serves as a powerful tool for improving the detection of both known and yet-to-emerge pathogens with zoonotic potential and relevance to emerging infectious diseases[100].

Our study quantifies the influence of tick species, geographical region, and host factors on tick microbiome composition. We observed significant variations in microbiomes among different tick genera within the same region and host species. Similarly, distinct microbiome profiles emerged within the same tick genus across different hosts in a given region, as well as across diverse regions for the same host-tick genus combination. These findings suggest that tick microbiome composition exhibits ecogeographical patterns[101]. Environmental factors such as temperature and humidity also play a crucial role in shaping tick microbiomes, indicating potential microbial adaptations to climate variability. For instance, a recent study comparing

the microbiomes of *I. scapularis* under different temperature conditions revealed that male ticks incubated at 37 ° C for 7-9 days exhibited a significant decline in Proteobacteria abundance, accompanied by a marked increase in Actinobacteria[102]. Our findings emphasize the combined influence of ecogeographical and genetic factors on tick microbiomes and provide valuable data for a deeper understanding of tick ecology and microbiota dynamics. Further investigations are needed to elucidate how environmental factors shape tick microbiota, which in turn influences tick behavior and disease transmission.

Ticks have evolved to host microbial endosymbionts that supplement essential nutrients lacking in the blood meals, such as B vitamins[33,37]. Currently, *Coxiella*-like endosymbionts, *Rickettsia*-like endosymbionts, *Francisella*-like endosymbionts, and *Candidatus* Midichloria mitochondrii are common symbionts in ticks, many of which can be transmitted to the next generation via transovarial transmission[35]. These intracellular symbionts play crucial roles in tick nutrition, reproduction, development, immunity, and environmental stress tolerance. For example, that the antibiotic-mediated removal of *Francisella* bacteria from ticks significantly reduces molting rates, impedes the development of nymphs into adults, and markedly decreases the weight of adult ticks[103]. Moreover, tick vector competence for pathogen transmission is influenced by symbionts. In *Dermacentor andersoni*, for example, *Rickettsia peacockii* hampers both the multiplication and transovarial transmission of the spotted fever agent, *Rickettsia rickettsii*[104].Our large-scale metagenomic analysis of tick microbiomes reveals that endosymbionts are ubiquitous across tick species but exhibit genus-specific associations, suggesting a more defined evolutionary relationship between endosymbionts and tick genera rather than individual species. For example, *Coxiella*-LE presents in *Haemaphysalis*, *Dermacentor*, *Ixodes*, but absent in *Amblyomma* and *Hyalomma*. Given that endosymbiont-based vector control strategies have been implemented at multiple sites to manage mosquito populations and reduce arbovirus transmission[105], the comprehensive survey and deeper understanding of tick symbioses could drive the development of novel control strategies targeting ticks and tick-borne diseases.

We identified 109 pathogen load-associated GO categories shared across all three tick species, encompassing biological processes such as tick development, metabolism, localization, cellular processes, reproduction, and stress response. This

finding highlights a connection between core biological functions essential for sustaining basic life activities in ticks and their pathogen-carrying capacity, while potentially suggesting how environmental selection pressures shape tick adaptation and pathogen transmission. Previous research in *R. microplus* indicated latitudinal clines in genomic structural variations[106], suggesting environmental influences on genome architecture. Our findings of stress response pathway mutations in all three tick species further support environmental impacts on tick adaptation. Our prior work suggested tick-specific ecological niche adaptation strategies[23], where such adaptations may simultaneously influence critical phenotypes including hematophagy efficiency and immune status, ultimately regulating pathogen loads. Collectively, our study suggests that environmentally driven natural selection can fix adaptive phenotypes in tick populations, thereby potentially modulating both tick evolution and pathogen transmission dynamics.

References for Supplementary Information

98. M. A. Jmel et al., Insights into the role of tick salivary protease inhibitors during ectoparasite-host crosstalk. Int J Mol Sci 22, (2021).

99. Y. Sun et al., A large-scale benchmark study of existing algorithms for taxonomy-independent microbial community analysis. Brief Bioinform 13,107–21 (2012).

100. K. K. K. Ko, K. R. Chng and N. Nagarajan. Metagenomics-enabled microbial surveillance. Nat Microbiol 7, 486–496 (2022).

101. X. B. Ni et al., Ecoclimate drivers shape virome diversity in a globally invasive tick species. ISEM J18, (2024).

102. S. Thapa, Y. Zhang, M. S. Allen, Effects of temperature on bacterial microbiome composition in *Ixodes scapularis* ticks. Microbiologyopen 8, e00719 (2019).

103. O. Duron et al., Tick-bacteria mutualism depends on B vitamin synthesis pathways. Curr Biol 28, 1896-1902.e1895 (2018).

104. S. I. Bonnet, F. Binetruy, A. M. Hernández-Jarguín, O. Duron, The tick microbiome: Why non-pathogenic microorganisms matter in tick biology and pathogen transmission. Front Cell Infect Microbiol 7, 236 (2017).

105. E. P. Caragata et al., *Wolbachia* as translational science: controlling mosquito-borne pathogens. Trends Parasitol. 37, 1050–1067 (2021).

106. Liu Q, et al., Mapping structural variations in Haemaphysalislongicornis and Rhipicephalus microplus reveals vector-pathogen adaptation. iScience 26, 106398

(2023).
