# Supplementary material for: Genome-resolved metagenomics reveals microbiome diversity across 48 tick species
Source: Nat Microbiol. 2025 Sep 23;10(10):2631–45. doi: 10.1038/s41564-025-02119-z (PMC12488481; doi:10.1038/s41564-025-02119-z)
Supplement: Supplementary file 2 — Reporting Summary [file 41564_2025_2119_MOESM2_ESM.pdf]

nature portfolio

Corresponding author(s): Wu-Chun Cao

Last updated by author(s): Aug 5, 2025

Reporting Summary

Nature Portfolio wishes to improve the reproducibility of the work that we publish. This form provides structure for consistency and transparency in reporting. For further information on Nature Portfolio policies, see our Editorial Policies and the Editorial Policy Checklist.

Statistics

For all statistical analyses, confirm that the following items are present in the figure legend, table legend, main text, or Methods section.

n/a	Confirmed	
☐	☒	The exact sample size (*n*) for each experimental group/condition, given as a discrete number and unit of measurement
☐	☒	A statement on whether measurements were taken from distinct samples or whether the same sample was measured repeatedly
☐	☒	The statistical test(s) used AND whether they are one- or two-sided<br>*Only common tests should be described solely by name; describe more complex techniques in the Methods section.*
☐	☒	A description of all covariates tested
☒	☐	A description of any assumptions or corrections, such as tests of normality and adjustment for multiple comparisons
☐	☒	A full description of the statistical parameters including central tendency (e.g. means) or other basic estimates (e.g. regression coefficient) AND variation (e.g. standard deviation) or associated estimates of uncertainty (e.g. confidence intervals)
☐	☒	For null hypothesis testing, the test statistic (e.g. *F*, *t*, *r*) with confidence intervals, effect sizes, degrees of freedom and *P* value noted<br>*Give P values as exact values whenever suitable.*
☒	☐	For Bayesian analysis, information on the choice of priors and Markov chain Monte Carlo settings
☒	☐	For hierarchical and complex designs, identification of the appropriate level for tests and full reporting of outcomes
☒	☐	Estimates of effect sizes (e.g. Cohen's *d*, Pearson's *r*), indicating how they were calculated

Our web collection on statistics for biologists contains articles on many of the points above.

Software and code

Policy information about availability of computer code

Data collection	Environment factor information was downloaded from National Geomatics Center of China (https://www.ngcc.cn/)
Data analysis	All software used in this study have been described in previously published articles. In summary, we employed software of Porechop v0.2.454, Nanofilt v2.6.055, Filtlong v0.2.0 , Flye v2.856, Racon v1.4.13, Pilon v1.23, NextPolish v1.3.0, Kraken2 v2.1.057, RepeatModeler v2.0.558, Trinity v2.4.059, PASA v2.3.360, Augustus v3.361, TBLASTN v2.2.28+62, Genomethreader v5.4.063, EvidenceModeler (EVM) v1.1.164, orthofinder v2.5.465, MUSCLE v3.8.155166, Gblocks v0.91b6, iqtree v2.2.068, r8s, AfterQC v0.9.670, Mitoz v3.671, bowtie2 v2.4.172, samtools v1.973, Megahit v1.2.974, SPAdes v3.15.275, MetaBAT2 v2.2.1576, CheckM v1.1.377, Prokka v1.14.678, fastANI v1.3227, Louvain v0.1681, python 3.10.0, PhyloPhlAn v3.0.6080, Vegan v2.6-281□phateR, Rtsne, A3 package, emapper v2.1.984, diamond v0.9.3586, BEDTools v2.27.188, SeqKit v2.3.089, GATK4 v4.0.12.090, BCFtools v0.1.1673, TASSEL v5.2.4092, GAPIT v3.2.093, SnpEff v5.295, PLINK v1.90b496 in this study.

For manuscripts utilizing custom algorithms or software that are central to the research but not yet described in published literature, software must be made available to editors and reviewers. We strongly encourage code deposition in a community repository (e.g. GitHub). See the Nature Portfolio guidelines for submitting code & software for further information.

Data

Policy information about availability of data

All manuscripts must include a data availability statement. This statement should provide the following information, where applicable:

- Accession codes, unique identifiers, or web links for publicly available datasets
- A description of any restrictions on data availability
- For clinical datasets or third party data, please ensure that the statement adheres to our policy

The genome assemblies for 19 tick species in this study are available at ENA: PRJEB89832 (https://www.ebi.ac.uk/ena/browser/view/PRJEB89832) and NGDC: PRJCA037692 (https://ngdc.cncb.ac.cn/bioproject/browse/PRJCA037692). The raw Illumina sequencing data are available at ENA: PRJEB89768 (https://www.ebi.ac.uk/ena/browser/view/PRJEB94929) and NGDC: PRJCA017096 (https://ngdc.cncb.ac.cn/bioproject/browse/PRJCA017096) and PRJCA002242 (https://ngdc.cncb.ac.cn/bioproject/browse/PRJCA002242). The genomes for 1373 rMAGs in this study are available at ENA: PRJEB89768 (https://www.ebi.ac.uk/ena/browser/view/PRJEB89768) and NGDC: PRJCA037694 (https://ngdc.cncb.ac.cn/bioproject/browse/PRJCA037694).

Research involving human participants, their data, or biological material

Policy information about studies with human participants or human data. See also policy information about sex, gender (identity/presentation), and sexual orientation and race, ethnicity and racism.

Reporting on sex and gender	Human participants were not involved in this study.
Reporting on race, ethnicity, or other socially relevant groupings	No socially relevant groupings was involved in this study.
Population characteristics	Population characteristics were not involved in this study.
Recruitment	Human participants recruitment were not involved in this study.
Ethics oversight	Human participants were not involved in this study.

Note that full information on the approval of the study protocol must also be provided in the manuscript.

Field-specific reporting

Please select the one below that is the best fit for your research. If you are not sure, read the appropriate sections before making your selection.

☒ Life sciences ☐ Behavioural & social sciences ☐ Ecological, evolutionary & environmental sciences

For a reference copy of the document with all sections, see nature.com/documents/nr-reporting-summary-flat.pdf

Life sciences study design

All studies must disclose on these points even when the disclosure is negative.

Sample size	This study represents the largest dataset on tick microbiomes to date. We conducted tick sampling across the nation as extensively as possible. In studying the species diversity and types of microbes carried by ticks using the specaccum, the species identified have reached saturation.
Data exclusions	No data were excluded from the analyses.
Replication	Replication of experiments were not involved in this study.
Randomization	Tick samples were pooled according to same species, same sex, same collection site and same blood-feeding status.
Blinding	Blinding was not relevant to this study, because since this study did not involve comparative research before and after treatment.

Reporting for specific materials, systems and methods

We require information from authors about some types of materials, experimental systems and methods used in many studies. Here, indicate whether each material, system or method listed is relevant to your study. If you are not sure if a list item applies to your research, read the appropriate section before selecting a response.

Materials & experimental systems

n/a	Involved in the study
☒	☐ Antibodies
☒	☐ Eukaryotic cell lines
☒	☐ Palaeontology and archaeology
☐	☒ Animals and other organisms
☒	☐ Clinical data
☒	☐ Dual use research of concern
☒	☐ Plants

Methods

n/a	Involved in the study
☒	☐ ChIP-seq
☒	☐ Flow cytometry
☒	☐ MRI-based neuroimaging

Animals and other research organisms

Policy information about studies involving animals; ARRIVE guidelines recommended for reporting animal research, and Sex and Gender in Research

Laboratory animals	The study did not involve laboratory animals.
Wild animals	Not applicable.
Reporting on sex	The samples in this study include both male and female ticks.
Field-collected samples	Ticks were collected from 31 provinces, metropolises or autonomous regions of mainland China. The collection sites were selected according to their ecological environments, including coniferous forest, steppe, farmland, desert, shrub-land and tropical forest. Ticks were collected by dragging a standard 1 m2 flannel flag over vegetation or from domestic or wild animals such as cattle, dogs, sheep, goats, cats, rabbits, camels, deer, and boars. Domestic animals were sampled from local farms, while wild animals were sourced from two origins: wildlife rescue centers and licensed breeding facilities. Ticks were collected with forceps from the animals after obtaining consent from the responsible personnel or owners.
Ethics oversight	The collection and removal of ticks are routine veterinary procedures and do not involve the acquisition of animal-derived materials. According to the Guideline for Ethical Review of Animal Welfare, no additional ethical approval is required for these procedures.

Note that full information on the approval of the study protocol must also be provided in the manuscript.

Plants

Seed stocks	*Report on the source of all seed stocks or other plant material used. If applicable, state the seed stock centre and catalogue number. If plant specimens were collected from the field, describe the collection location, date and sampling procedures.*
Novel plant genotypes	*Describe the methods by which all novel plant genotypes were produced. This includes those generated by transgenic approaches, gene editing, chemical/radiation-based mutagenesis and hybridization. For transgenic lines, describe the transformation method, the number of independent lines analyzed and the generation upon which experiments were performed. For gene-edited lines, describe the editor used, the endogenous sequence targeted for editing, the targeting guide RNA sequence (if applicable) and how the editor was applied.*
Authentication	*Describe any authentication procedures for each seed stock used or novel genotype generated. Describe any experiments used to assess the effect of a mutation and, where applicable, how potential secondary effects (e.g. second site T-DNA insertions, mosiacism, off-target gene editing) were examined.*
